# Evaluation of RNA Interference for Control of the Grape Mealybug *Pseudococcus maritimus* (Hemiptera: Pseudococcidae)

**DOI:** 10.3390/insects11110739

**Published:** 2020-10-28

**Authors:** Arinder K. Arora, Noah Clark, Karen S. Wentworth, Stephen Hesler, Marc Fuchs, Greg Loeb, Angela E. Douglas

**Affiliations:** 1 Department of Entomology, Cornell University, Ithaca, NY 14850, USA; aka76@cornell.edu (A.K.A.); clark.noah@gmail.com (N.C.); 2 Department of Entomology, Cornell University, Geneva, NY 14456, USA; ksw2@cornell.edu (K.S.W.); sph12@cornell.edu (S.H.); gme1@cornell.edu (G.L.); 3 School of Integrative Plant Science, Cornell University, Geneva, NY 14456, USA; marc.fuchs@cornell.edu; 4 Department of Molecular Biology and Genetics, Cornell University, Ithaca, NY 14853, USA

**Keywords:** grapevine pests, mealybugs, osmoregulation genes, phloem-feeding insects, *Pseudococcus maritimus*, RNA interference

## Abstract

**Simple Summary:**

RNA interference (RNAi) is a defense mechanism that protects insects from viruses by targeting and degrading RNA. This feature has been exploited to reduce the expression of endogenous RNA for determining functions of various genes and for killing insect pests by targeting genes that are vital for insect survival. When dsRNA matching perfectly to the target RNA is administered, the RNAi machinery dices the dsRNA into ~21 bp fragments (known as siRNAs) and one strand of siRNA is employed by the RNAi machinery to target and degrade the target RNA. In this study we used a cocktail of dsRNAs targeting grape mealybug’s aquaporin and sucrase genes to kill the insect. Aquaporins and sucrases are important genes enabling these insects to maintain water relations indispensable for survival and digest complex sugars in the diet of plant sap-feeding insects, including mealybugs. In our experiments, administration of dsRNA caused a reduction in expression of the target genes and an increase in insect mortality. These results provide support for the application of RNAi to control the grape mealybug.

**Abstract:**

The grape mealybug *Pseudococcus maritimus* (Ehrhorn, 1900) (Hemiptera: Pseudococcidae) is a significant pest of grapevines (*Vitis* spp.) and a vector of disease-causing grape viruses, linked to its feeding on phloem sap. The management of this pest is constrained by the lack of naturally occurring resistance traits in *Vitis*. Here, we obtained proof of concept that RNA interference (RNAi) using double-stranded RNA (dsRNA) molecules against essential genes for phloem sap feeding can depress insect survival. The genes of interest code for an aquaporin (*AQP*) and a sucrase (*SUC*) that are required for osmoregulation in related phloem sap-feeding hemipteran insects (aphids and whiteflies). In parallel, we investigated the grape mealybug genes coding non-specific nucleases (*NUC*), which reduce RNAi efficacy by degrading administered dsRNA. Homologs of *AQP* and *SUC* with experimentally validated function in aphids, together with *NUC*, were identified in the published transcriptome of the citrus mealybug *Planococcus citri* by phylogenetic analysis, and sequences of the candidate genes were obtained for *Ps. maritimus* by PCR with degenerate primers. Using this first sequence information for *Ps. maritimus*, dsRNA was prepared and administered to the insects via an artificial diet. The treatment comprising dsRNA against *AQP*, *SUC* and *NUC* significantly increased insect mortality over three days, relative to dsRNA-free controls. The dsRNA constructs for *AQP* and *NUC* were predicted, from sequence analysis to have some activity against other mealybugs, but none of the three dsRNA constructs have predicted activity against aphids. This study provides the basis to develop in planta RNAi strategies against *Ps. maritimus* and other mealybug pests of grapevines.

## 1. Introduction

The grape mealybug, *Pseudococcus maritimus* (Ehrhorn, 1900), is a polyphagous insect that feeds through its life cycle on plant phloem sap. It has a broad geographical distribution in Eurasia and North America, and, throughout its range, it is a pest of deciduous fruit crops, including apple, pear and grape [1]. In particular, *Ps. maritimus* reduces the vigor of grape plants by direct feeding damage, the deposition of honeydew and by transmission of grapevine leafroll-associated viruses (GLRaVs), reducing grape yield by up to 70% [2,3,4].

*Ps. maritimus* can be controlled by systemic insecticides [5]. It is unclear, however, whether conventional insecticidal control, on its own, is sufficiently effective to reduce the spread of GLRaVs [4]. Therefore, this pest is an important target for the development of alternative control strategies. A promising candidate approach is RNA interference (RNAi)-mediated suppression of the expression of essential insect genes, which can be targeted specifically to the pest species with no deleterious effects on other organisms, including beneficial insects and the human consumer [6,7,8,9]. This specificity arises from the molecular mechanism of RNAi [10,11]. Briefly, double-stranded RNA (dsRNA) is internalized into cells, where it is cut by an enzyme, Dicer, into 21 bp fragments known as small interfering RNA duplex (siRNA duplex). The siRNA duplex is then loaded onto the multi-subunit RNA-induced Silencing Complex (RISC) complex, one strand of the siRNA is selected and any mRNA with complementary sequence is degraded by the enzyme Argonaute. The dsRNA can be delivered to insects by feeding, either as a food supplement or, for phytophagous insects, by genetic modification of the plant [12,13,14]. Transcriptome data [15,16] demonstrate that RNAi is active in the citrus mealybug *Planococcus citri* (Risso, 1900) (Hemiptera: Pseudococcidae) and the cotton mealybug *Phenacoccus solenopsis* (Tinsley, 1898) (Hemiptera: Pseudococcidae), and RNAi has been successfully applied to these two species [17,18,19].

The basis for our strategy to develop RNAi as a control strategy against *Ps. maritimus* was the prior research on RNAi against other phloem-feeding hemipteran insects of the suborder Sternorrhyncha. Sternorrhyncha comprises the Coccoidea (scale insects and mealybugs), Aleyrodoidea (whiteflies), Psylloidea (psyllids) and Aphidomorpha (aphids and phylloxerids). Our specific focus is the genes encoding aquaporin water channel (*AQP*) and sucrase (*SUC*), which are overly expressed in the gut and their translated proteins mediate the osmoregulatory control of body fluids of these insects [20,21,22]. Specifically, the sugar-rich diet of phloem sap exerts a high osmotic pressure that, on ingestion into the gut, is predicted to draw water from the insect body fluids to the gut lumen. The loss of body water leads to insect desiccation and death [23,24,25]. Osmotic collapse is prevented by two enzymatic activities of the sucrase; first, sucrase-mediated hydrolysis of ingested sucrose and, second, transglucosidation of the released glucose to form oligosaccharides, thereby reducing the osmotic pressure, together with aquaporin-facilitated water cycling between the distal and proximal gut regions [26]. *AQP* and *SUC* have been validated experimentally in the pea aphid *Acyrthosiphon pisum* (Harris, 1776) (Hemiptera: Aphididae) [21,22] and *AQP* in the whitefly *Bemisia tabaci* (Gennadius, 1889) (Hemiptera: Aleyrodidae) [20,27]. Furthermore, RNAi knockdown of the expression of one or both *AQP* and *SUC* increases mortality in the aphid *Myzus persicae* (Sulzer, 1776) (Hemiptera: Aphididae), the whitefly *Bemisia tabaci*, the mealybug *Ph. solenopsis* and the psyllid *Diaphorina citri* (Kuwayama, 1908) (Hemiptera: Liviidae) [16,28,29,30,31]. We reasoned that this technology would be applicable to *Ps. maritimus*, following phylogenetic evidence that the citrus mealybug *Pl. citri* has genes homologous to the osmoregulatory *AQP* and *SUC* in aphids and whiteflies [32].

The goal of this study was to obtain proof of principle that RNAi can be applied against *Ps. maritimus.* This required us to address three challenges. The first was to develop a reliable protocol for laboratory culture of the insect for experimental study, and a feeding protocol for dsRNA delivery. The second was to obtain *AQP* and *SUC* sequences for dsRNA synthesis. No published sequence data for this species are available, and our strategy was to sequence PCR amplicons generated from *Ps. maritimus* cDNA with degenerate primers designed from homologous genes in other insects. The final challenge arose from the well-documented variability in RNAi efficacy against phloem-feeding insects and other insects [7,33,34,35]. Focusing on RNAi against osmoregulation genes in phloem-feeding insects, research on aphids and whiteflies has revealed that RNAi efficacy is markedly improved by supplementing the administered ds*AQP* and ds*SUC* with RNAi against the gene coding a gut nuclease (*NUC*) that mediates the nonspecific degradation of ingested dsRNA [28,36,37]. The dsRNA-degrading activity has also been reported in the mealybug *Ph. solenopsis*, together with evidence that dsRNA against the nuclease sequence improves RNAi efficacy, but the repertoire of candidate *NUC* in mealybugs has not been explored systematically [16]. Anticipating that nuclease activity may impair RNAi against *Ps. maritimus*, our molecular analysis of this species was extended to identify *NUC* sequences, and then to co-administer the dsRNA against *AQP, SUC* and *NUC* to the insects. This study reports the development of specific RNAi molecules for a pest species that lacked prior molecular information, and it provides the first evidence for RNAi against osmoregulation genes as a potential control approach against *Ps. maritimus*.

## 2. Materials and Methods

### 2.1. Insects

Grape mealybugs *Ps. maritimus* were collected from a vineyard (*V. vinifera* cultivar Chardonnay) in Seneca County, Finger Lakes region of NY (42°41′05.38″ N and 76°44′37.52″ W) on 19 and 25 July and 2 August 2018, and combined to generate a laboratory culture on *V. vinifera* cv. “Pixie” [38]. The culture was maintained at 21 °C with 17 h light:7 h dark. Frozen vine mealybugs *Pl. ficus* were provided by Dr. Kent Daane from a culture at The Kearney Agricultural Research and Extension Center, Parlier, CA, derived from collections in Fresno County, CA.

### 2.2. RNA Extraction

For each gut sample, 30–35 insects were dissected in diethyl pyrocarbonate (DEPC)-treated phosphate-buffered saline (PBS), pH 7.4, using fine pins under a dissecting microscope at 1.5-2X magnification. The isolated guts were immediately transferred to 50 µL RNAlater (Thermo Fisher, Waltham, MA, USA), stored at 4 °C overnight, and then at −20 °C. Immediately prior to RNA extraction, 75 µL DEPC-treated PBS were added to each thawed tube, mixed gently by inverting 10 times, and centrifuged at 5000 *g* for 5 min. The supernatant was removed.

Each sample of whole bodies or pelleted guts of *Ps. maritimus*, and each whole body sample of *Pl. ficus* was mixed with 200 µL or 50 µL RNAzol (catalog # R4533, Millipore Sigma, Burlington, MA, USA), respectively, and then homogenized using a plastic pestle. An additional 150 µL RNAzol was added to the gut homogenates, which were then vortexed. Total RNA was extracted following the manufacturer’s RNAzol RT protocol. The RNA was precipitated with an equal amount of cold isopropanol and 1 µL linear acrylamide. Following incubation, for 10 min at room temperature (whole bodies) or overnight at −20 °C (guts), the samples were centrifuged at 12,000× *g* for 10 min at 4 °C. The pellet was washed twice with ice-cold 75% ethanol and resuspended in 15 µL nuclease-free water.

### 2.3. Design of Primers for Amplification of Ps. maritimus and Pl. ficus Genes

The published phylogenetic trees for the three genes of interest, *AQP1*, *SUC1* (32) and *NUC1* (28) in sternorrhynchan Hemiptera were updated with additional sequence data recently published in NCBI, including sequences of the mealybug *Pl. citri*, as described in Text S1 and displayed in Figures S1–S3. In parallel, sequences were collated for the *β-tubulin* gene (Figure S4) used as a normalizing gene for quantitative PCR. The sequences for each gene were aligned using the multiple sequence alignment tool MUSCLE [39] (www.ebi.ac.uk/Tools/msa/muscle/), in conjunction with the Base-by-Base editor (4virology.net/virology-ca-tools/base-by-base), to identify conserved sequences. Degenerate primers were then designed using Primaclade (http://primaclade.org/cgi-bin/primaclade.cgi) (Table S1A).

### 2.4. cDNA Synthesis and Sequencing

First-strand cDNA was synthesized using a Superscript II Reverse Transcriptase Kit (Catalog # 18064014, ThermoFisher Scientific, Waltham, MA, USA) and 100 ng RNA from whole bodies of *Ps. maritimus* or *Pl. ficus*, following the manufacturer’s instructions. The cDNA was then used as a template for amplification of the predicted orthologs of *AQP1*, *SUC1* and *NUC1*, using 12.5 μL GoTaq Green Master Mix (Catalog # M7122, Promega, Madison, WI, USA), 0.625 μM of each primer (Supplemental Table S1A), 1 μL cDNA template and 9 μL water. The PCR reaction conditions were optimized for each gene (Supplemental Table S2). The PCR products were purified using a QIAquick PCR Purification Kit (Catalog # 28104, Qiagen, Hilden, Germany) following the manufacturer’s instructions and checked for predicted length by gel electrophoresis, prior to Sanger sequencing. The sequences are available at NCBI as accession numbers MT187985, MT187986, MT187988, MT187989, MT192030, MT192031 and MT192032.

### 2.5. Quantitative Real-Time PCR (qRT-PCR)

Gene expression was quantified by qRT-PCR that comprised 5 µl iQ SYBR Green supermix (Catalog# 1708862, BioRad, Hercules, CA), 0.5 µl primers, each at 10 µM (Table S1B), 1 µL cDNA and 3 µL water. The reactions were run in a C100 Thermal Cycler with a CFX96 Touch^TM^ Real-Time PCR Detection System (Bio-Rad) at 95 °C for 3 min, 95 °C for 10 s, followed by annealing and extension at 60 °C for 30 s for 40 cycles. A dissociation curve included in each assay comprised 65 °C to 95 °C (minimum to maximum temperatures) in 0.5 °C increments per 0.05 s, yielding single gene-specific peaks. All experiments included *Ps. maritimus β-tubulin* (accession number MT187989) as a normalizing gene and template-free samples as negative controls. The mean *Ct* values for two technical replicates were calculated, and the difference between experimental and control samples was determined as the log_2_ fold difference [40].

### 2.6. Synthesis of dsRNA

A target sequence for each gene was identified using SOL Genomics Network VIGStool (www.solgenomics.net) with no predicted non-target activity against *Vitis vinifera.* Each sequence—ds*AQP1* (250 bp), ds*SUC1* (240 bp) and ds*NUC1* (250 bp)—was amplified from *Ps*. *maritimus* cDNA (primers provided in Table S1C), cloned in pGEMT Easy plasmid (Catalog # A1360, Promega, Madison, WI, USA), and transformed into *Escherichia coli* DH5α (Catalog # 18258012, ThermoFisher Scientific), following the manufacturer’s protocol. The cloned gene was sequenced to confirm the sequence. The plasmid was purified using a Zymo PURE Plasmid Miniprep Kit (Catalog # D4209, Zymo Research, Irvine, CA, USA), and the target sequence was confirmed by Sanger sequencing. The target sequence was amplified and used as a template for dsRNA synthesis by an AmpliScribe T7-Flash Transcription Kit (Catalog # ASF3507, Lucigen, Middleton, WI, USA), and purified following the manufacturer’s instructions. The size of synthesized dsRNA was confirmed by gel electrophoresis, and dsRNA concentration was measured using a NanoDrop photometer. In parallel, a plasmid pGFP2 containing a 370 bp long green fluorescent protein (GFP) DNA template [28] was used to synthesize dsRNA of GFP.

### 2.7. RNAi Experiments

**Figure 1:**
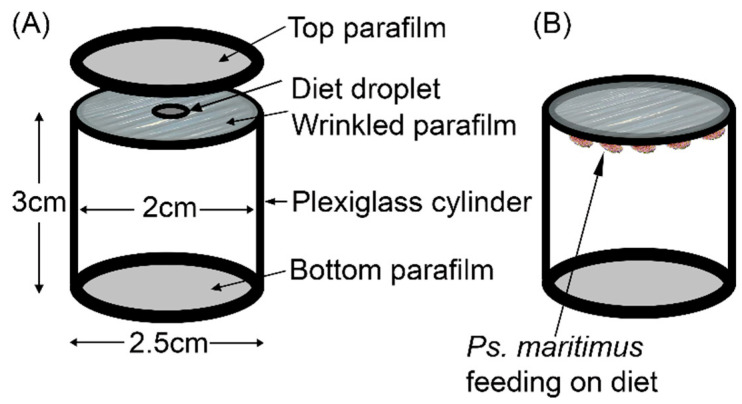
Rearing *Pseudococcus maritimus* on an artificial liquid diet. (**A**) The components of the diet cage, with diet sachet at the top. (**B**) An assembled diet cage with feeding *P. maritimus*.

dsRNA was delivered orally to ca. 30-day-old (2nd–3rd instar) *Ps. maritimus* mealybugs, using a sachet of 100 µL sterile artificial liquid diet sandwiched between two layers of Parafilm. The method followed the procedure previously developed for aphids [41], with two modifications (Figure 1). First, the Parafilm diet sachet was not stretched taut, following observations that mealybugs preferentially feed at the wrinkles in the Parafilm. Second, the base of the cage was sealed with a single sheet of Parafilm, after the addition of the insects to the cage, to prevent escape. Each 5-day experiment comprised a single replicate cage for five treatments, and the experiment was repeated four times over four consecutive weeks. The experimental design comprised a 2-day pre-treatment and a 3-day treatment (Table 1). The pre-treatment diets were supplemented or not with ds*NUC1*. For the treatment phase of the experiment, the test diets contained ds*AQP1* and ds*SUC1*, either with or without ds*NUC1* (treatments A+S and N+A+S, respectively). As controls for non-specific effects of dsRNA, replicated sets of insects were fed with **ds*GFP*** (treatment G) or ds*GFP* and dsNUC1 (treatment N+G), and the experiments also included dsRNA-free diet (treatment Diet). The ds*NUC1* was delivered at 0.2 µg µL^−1^, and the total dsRNA concentration was 0.4 µg µL^−1^ in all treatments at day 2 to day 5 (see Table 1 for details). On day 5 of the experiment (Table 1), the surviving insects were counted, and the guts were dissected and stored in RNAlater for RNA extraction and quantification of gene expression by qRT-PCR.

### 2.8. Species Specificity of dsRNA Reagents

The predicted activity of dsRNA sequences designed for *Ps. maritimus* against *Pl. citri* and *Pl. ficus* was investigated using siRNA-Finder (si-Fi) software (https://github.com/snowformatics/siFi21) [42], using default settings with 21 bp siRNA and zero mismatch. The cDNA sequences of *AQP1*, *NUC1* and *SUC1* of non-target species were added in si-Fi as the database and dsRNA sequences of *AQP1*, *NUC1* and *SUC1* from *Ps. maritimus* were used as a query to find the numbers of 21 bp segments that matched perfectly.

### 2.9. Statistical Analysis

The data were analyzed using R software version 3.5.1 [43]. To analyze mealybug survival, a generalized linear model with a logit link was performed using the “glmer” function from the lme4 package version 1.1–2.1, with dsRNA treatments as categorical fixed effects and experiment as a random effect [43,44]. The log_2_ fold differences in gene expression were analyzed as a linear model using the “lm” function, with treatments as categorical predictors. To test for differences in mealybug survival and gene expression between insects on the ds*GFP* diet (treatment G in Table 1) and other treatments, the regression coefficient for each treatment was calculated using the coefficient regression *t*-test in the “glmer” and “lm” functions with treatment G set as the reference.

## 3. Results

### 3.1. Identification of Target Genes in Pseudococcus maritimus and Design of dsRNA Sequences

At the start of this study, no sequence information was available for the mealybug *Ps. maritimus*, but a single ortholog of the osmoregulation genes of the pea aphid (*ApAQP1* and *ApSUC1*) had been identified in the transcriptome of a different mealybug species, *Planococcus citri* [32]. Our first analysis made use of recently published sequence data to update the phylogeny of *AQP* genes and glycoside hydrolase family 13 (including *ApSUC1*) genes in the sternorrhynchan hemipterans (Figures S1A,B and S2A,B). The consensus Bayesian and maximum likelihood (ML) trees confirmed the single orthologs of *ApAQP1* and *ApSUC1* in *Pl. citri*, and we named these orthologs *PcAQP1* and *PcSUC1*, respectively. Applying the same approach, we investigated homolog(s) of the pea aphid nuclease (*ApNUC1*), which mediates the nonspecific degradation of orally delivered dsRNA [37]. The Bayesian and ML analyses (Figure S3A,B) identified a single ortholog in *Pl. citri*, which we named *PcNUC1*.

We then designed degenerate primers to regions of *AQP1*, *SUC1* and *NUC1* that were predicted not to amplify other aquaporin, GH-13 or nuclease genes, based on the gene sequences of the mealybug *Pl. citri*, the aphids *A. pisum* and *M. persicae* and the whitefly *B. tabaci* (Figures S1C, S2C and S3C; Table S1A). With these primers and end-point PCR reactions, we amplified orthologous genes from cDNA of our focal mealybug species, *Ps. maritimus*. Sanger sequencing of the PCR products yielded single sequences that had high sequence similarity to *PcAQP1*, *PcSUC1* and *PcNUC1* and that, on translation, generated the predicted protein domains using the NCBI conserved domain database (https://www.ncbi.nlm.nih.gov/cdd/). The *Ps. maritimus* sequences are available in NCBI as accession numbers MT187985, MT187986 and MT187988, respectively.

Finally, we designed dsRNA sequences against each of the three *Ps. maritimus* genes of interest and cloned them as described in the Material and Methods. Because dsRNA can potentially be used to develop pest control strategies, we tested the predicted non-target potential of the dsRNAs against homologous gene sequences of insects other than *Ps. maritimus* (Table 2). Our results revealed that *dsAQP1* and ds*NUC1* have limited predicted non-target potential to other mealybug species of the same family (detailed in Figure S5), and no predicted non-target potential against species in the sister group, the aphids; *dsSUC1* has no predicted potential against either mealybug or aphid species.

### 3.2. Expression of Target Genes in the Gut of Ps. maritimus

**Figure 2:**
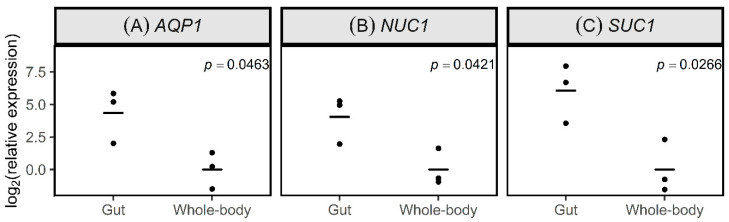
Expression of genes in the gut and whole body of 2nd–3rd instar nymphs of *Pseudococcus maritimus*. The mean values are shown as horizontal bars. Target genes are (**A**) *AQP1*, (**B**) *NUC1* and (**C**) *SUC1*. Three biological replicates, each comprising 30–35 guts and 10–15 whole bodies were used. The data were analyzed by *t*-test, and p-values are provided.

We quantified the expression of the target genes *PmAQP1*, *PmSUC1* and *PmNuc1* in dissected guts, compared to whole bodies, for three separate collections from the laboratory colony of *Ps. maritimus* (Figure 2). Transcripts of all three genes were significantly more abundant in the gut samples, and the mean enrichment was 20-fold, 66-fold and 17-fold, respectively. These results are consistent with their putative function in the gut and their suitability for targeting via orally delivered RNAi.

### 3.3. Response of Ps. maritimus to Orally Delivered RNAi

**Figure 3:**
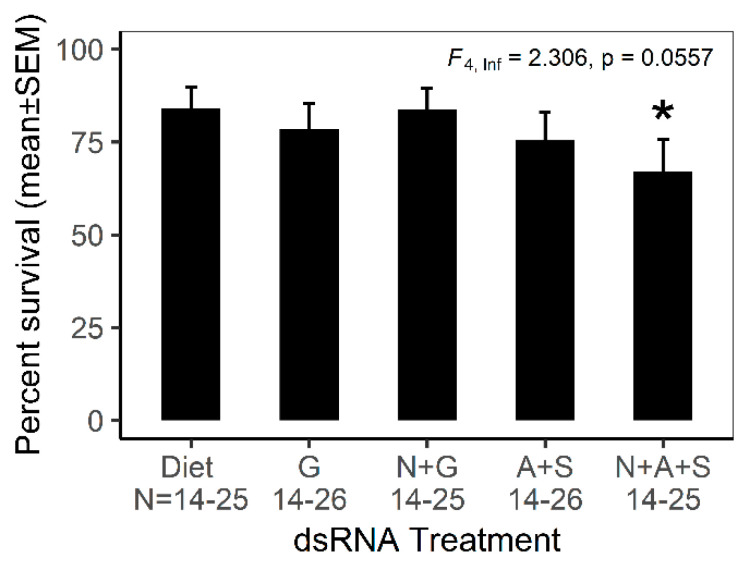
Impact of RNAi on survival of *Ps. maritimus* over 5 days. GLMER test with critical probability of 0.1 was applied to compare each treatment to the dsRNA-free diet control, and significantly different treatment is indicated by asterisk (results from statistical analysis are provided in Table S3). The experiment was conducted four times, each time with a single diet cage per treatment. The range of the number of insects per replicate for each treatment (N) is shown on the *x*-axis. The acronyms for treatments are: A, ds*AQP1*; G, ds*GFP*; N, ds*NUC1*; S, ds*SUC1* (see Table 1).

In preliminary experiments, we administered dsRNA to the mealybugs by two complementary methods. The first was the petiole dip assay, i.e., the petiole of a freshly cut grape leaf bearing feeding mealybugs was immersed in a solution of dsRNA [16]. However, the insects failed to settle and feed on the excised leaves, whether the petiole was immersed in deionized water or dsRNA preparations. This method was not pursued further. Our second approach used membrane feeding with an artificial liquid diet sandwiched between two layers of Parafilm. The mealybugs settled and fed from the artificial diet, especially favoring the edges of folds that we mechanically introduced to the Parafilm membrane (Figure 1). The insects could be maintained on the artificial diet for up to one week with low mortality. Long-term culture of *Ps. maritimus* on the artificial diet was not attempted because transfers to a fresh diet without damaging the insects was inordinately time-consuming. Based on our preliminary trials, we devised a 5-day experimental protocol (Table 1) using mealybugs administered dsRNA via the artificial diet. On the dsRNA-free diet (‘Diet’ in Table 1), 80% of the mealybugs survived the 3-day treatment (Figure 3 and Dataset S1). Survival varied significantly across the five treatments. Compared to treatment G (dsRNA against GFP), the linear model yielded a statistically significant reduction in survival of treatment N+A+S (dsRNA against *PmAQP1*, *PmSUC1* and *PmNUC1*) (Figure 3 with statistical analysis in Table S3, *p* < 0.1). The non-significant effect of ds*AQP1* and ds*SUC1* without ds*NUC1* (treatment A+S) suggested that dsRNA against the nuclease gene promotes the efficacy of dsRNA against *PmAQP1* and *PmSUC1*, as previously demonstrated for aphids [37] and whiteflies [28].

**Figure 4:**
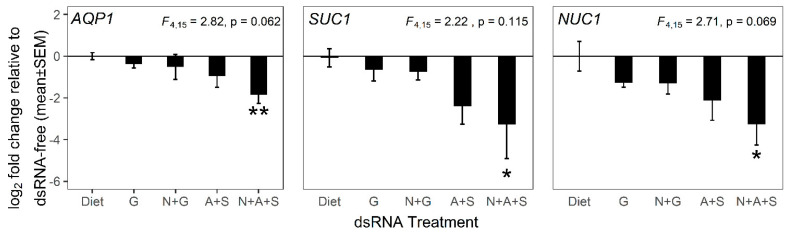
Impact of RNAi on expression of targeted *Ps. maritimus* genes *AQP1* (left panel), *SUC1* (middle panel) and *NUC1* (right panel). Linear model statistics were calculated and all the treatments were compared to dsRNA-free diet control. The double and single asterisks depict significant differences from dsRNA-free diet at α = 0.05 and 0.1, respectively (results from statistical analysis are provided in Table S4). The experimental details and treatment description are as in Figure 3. The range of number of insects used in each treatment are: Diet, 12–21; G, 10–18; N+G, 10–20; A+S, 9–19; N+A+S, 9–17.

We also quantified expression of *PmAQP1*, *PmSUC1* and *PmNUC1* in the guts dissected from insects that survived the 5-day experiment. Expression of all three genes varied significantly with treatment, and transcript abundance was significantly reduced in insects from treatment N+A+S relative to the GFP-dsRNA treatment (treatment G) (Figure 4 with statistical analysis in Table S4, *p* < 0.1). The data fitted to the expectation that dsRNA against *PmAQP1* and *PmSUC1* would result in reduced expression of these genes, respectively. However, *PmNUC1* expression was, similarly, expected to be reduced to an equivalent extent in the treatments that included ds*NUC1* (treatments N+G and N+A+S), but this result was not obtained. We discuss possible reasons for these results in the discussion.

## 4. Discussion

In this study, we obtained evidence that orally administered RNAi against osmoregulation genes causes a significant increase in mortality of the grape mealybug *Ps. maritimus* over a three-day period.

The rationale for this study arose from the generality that homologous *AQP* and *SUC* genes mediate osmoregulation in the gut of different phloem-feeding sternorrhynchan Hemiptera. This generated the expectation that candidate gene targets for a previously unstudied species can be identified by phylogenetic analysis of the aquaporin gene family and glucosyl hydrolase-13 (GH-13) gene family, respectively [27,32]. Consistent with this expectation, the candidate *Ps. maritimus* genes that we identified from phylogenetic analysis displayed the predicted features of osmoregulation genes: enriched expression in the gut and increased mortality of insects administered dsRNA specific to the sequence of these genes. However, definitive elucidation of homologies of osmoregulation genes must await sequencing of the *Ps. maritimus* genome to obtain the full set of aquaporin genes and GH-13 genes in this insect, and experimental validation of water transport and sucrase activity of the selected genes.

Novel methods to control insect pests tend to be developed on species that are amenable to laboratory cultivation. In this respect, *Ps. maritimus* poses major logistical problems because its availability in the field is seasonal and, prior to the husbandry advances that made this study possible, it failed to thrive under insectary conditions. Nevertheless, insect material was always limiting for this study, and our experimental designs depended on prior knowledge gained of other phloem-feeding insects. In particular, we made use of the evidence from published studies that RNAi against *AQP* and *SUC* genes can function synergistically [28,31], presumably because the dual RNAi reduces the capacity of each gene product to compensate for functional deficiencies in the other. These studies revealed that insects administered both ds*AQP1* and ds*SUC1* cannot compensate for the increased osmotic pressure in the gut caused by reduced transglucosidation (SUC1 function) by increased water cycling (AQP1 function), and *vice versa*. Further research conducted with larger numbers of insects will be required to establish the individual contributions of ds*AQP1* and ds*SUC1* and synergistic interactions between these treatments in RNAi against *Ps. maritimus.*

In our experiments, RNAi against the osmoregulation genes *AQP1* and *SUC1* yielded significantly increased mortality, relative to treatment G control, only in conjunction with the nuclease gene *NUC1*, which we demonstrated to have enriched expression in the gut (Figure 2). These results are fully consistent with the accumulating evidence that gut nuclease activity is a major cause of poor RNAi efficiency in various insects [28,36,37,45,46,47,48,49,50,51]. The function of the nuclease of sternorrhynchan hemipterans has not been investigated, other than as an inhibitor of RNAi. It is not an essential enzyme, in the sense that RNAi against this gene does not increase insect mortality ([28,37,52] and this study), although reduced nymphal growth of ds*NUC-*treated pea aphids has been reported [37]. The natural substrate of insect gut nucleases may be DNA and RNA in disrupted insect cells sloughed off to the gut lumen during the natural process of gut epithelium turnover [53,54], as well as nucleic acids in the phloem sap diet [55]. If the nucleotide products of gut nuclease activity are assimilated across the insect gut wall and used as substrates for *de novo* nucleic acid synthesis, the nuclease would have a nutritional role. However, our experiments cannot exclude the alternative explanation that *dsNUC1* pretreatment activated a nonspecific RNAi response. Such responses have been reported in other insects, e.g., [56,57], but have not been investigated in mealybugs. Further research can investigate this possibility, for example by including dsGFP control treatments in the pretreatment phase of experiments.

From the applied perspective of insect pest control, the effects of RNAi on insect performance is the prime criterion of RNAi efficacy. The complementary index of reduced expression of the target gene is widely considered to offer validation of mechanism [33,58]. The reduced transcript abundance of *AQP1* and *SUC1* in the guts of insects fed on diets containing these dsRNAs (treatments A+S and N+A+S in Table 1) supports this interpretation, but two aspects of the gene expression dataset (Figure 4) are inconsistent. First, we expected the expression knockdown of these target genes, relative to the ds*GFP*-containing diet (treatment G), to be greater in treatment A+S, with *dsAQP1* and *dsSUC1* at double the concentration in treatment N+A+S, but we obtained the opposite results. Second, the expression of *NUC1* is expected to be equivalent in treatments N+G and N+A+S, with administered *dsNUC1* at the same concentration. Instead, the expression of *dsNUC1*, ds*AQP1* and ds*SUC1* was reduced in treatment N+A+S, with the highest mortality. We hypothesize that reduced gene expression was, at least partly, a consequence (not a cause) of suboptimal performance, specifically because the living insects feeding on dsRNA against osmoregulation genes were unhealthy and displaying reduced expression of certain genes with gut-specific function relative to house-keeping genes. This interpretation is consistent with reports of weak or variable correspondence between transcript abundance and insect performance [31,33,37,59]. Importantly, our results for *NUC1* gene expression in ds*NUC1*-treated insects are very similar to published data for the nuclease gene in the sweet potato weevil *Cylas puncticollis* [60], where nuclease expression was decreased only when nuclease dsRNA was administered in conjunction with dsRNA against the target gene (specifically, *Snf7*). An important implication of this interpretation is that RNAi against the osmoregulation genes of *Ps. maritimus* would likely yield greater mortality effects over a longer time course than the three days adopted in this study. Such analysis, however, will require the development of *in planta* delivery of RNAi because the artificial diet system used here is unsuitable for extended experiments.

A further potential limitation of our expression analysis was that a single gene, *β-tubulin*, was used for normalization in the qPCR analyses. Although other candidate housekeeping genes were considered, none, other than *β-tubulin*, yielded a reliable sequence with degenerate primers. This is a consequence of the lack of genomic data for *Ps. maritimus*, and other expression analyses of mealybug species have, similarly, used a single gene for normalization [16,19]. Although the precision and accuracy of expression analyses are improved by the use of two or more housekeeping genes, two considerations indicate that our use of *β-tubulin* did not introduce substantial bias. First, *β-tubulin* is a reliable housekeeping gene for expression analysis in related hemipteran insects (whiteflies and aphids) [28,37]. Second, the elevated expression of the genes of interest for this study in the gut relative to the whole body (Figure 2) could not be attributed to reduced *β-tubulin* expression in the gut because expression of a further gene, *SUC4*, was not elevated in the gut (Arora, unpub. data). As additional molecular data become available for *Ps. maritimus*, it will become feasible to employ multiple reference genes for expression analysis.

A final issue raised by this study relates to the specificity of the RNAi treatments to the pest species. The predicted non-target potential of the dsRNA constructs used in this study against insects other than *Ps. maritimus* was limited to other mealybugs and did not extend to aphids, members of the sister group to Coccoidea. In the context of the grapevine system, the activity of single dsRNA constructs against all grape-infesting mealybugs is an efficient route for crop protection. These results should, however, be interpreted with great caution. The weak correspondence between gene expression levels and insect performance (see previous paragraph) is most readily explained by a contribution of perturbed translation of the target gene to the RNAi effect [61]. Importantly, lower sequence identity between the siRNA and target transcript is required for translational suppression than for the canonical RNAi mechanism of transcript degradation [62,63], with the implication that the dsRNA may have lower species specificity than predicted. Applying the principles of studies on the effects of RNAi triggers to nontarget insect species [9,64], a priority for future research is to determine empirically the efficacy of dsRNAs designed for *Ps. maritimus* against other grapevine mealybug pest species and other insects in the grapevine cultivation system.

## 5. Conclusions

In conclusion, this study provides the first demonstration of the feasibility of an RNAi-based strategy targeting insect osmoregulatory function for the control of the grapevine pest, *Ps. maritimus*. It offers the basis for targeted *in planta* analysis of RNAi against osmoregulation genes and, more generally, illustrates the opportunity to develop RNAi against insect pests that are traditionally perceived as intractable because they lack genomic resources and routine laboratory maintenance protocols.

## Figures and Tables

**Table 1:** Experimental design for RNA interference (RNAi) experiments.

Treatment	Double Stranded RNA (dsRNA) in Diet	
	Day 0 to Day 2 Larvae	Day 2 to Day 7 Larvae
Diet	dsRNA free	dsRNA free
G	dsRNA free	ds*GFP* (0.4 µg µL^−1^)
N+G	ds*NUC1* (0.2 µg µL^−1^)	ds*GFP* (0.2 µg µL^−1^) + ds*NUC1* (0.2 µg µL^−1^)
A+S	dsRNA free	ds*AQP1* (0.2 µg µL^−1^) + ds*SUC1* (0.2 µg µL^−1^)
N+A+S	ds*NUC1* (0.2 µg µL^−1^)	ds*AQP1* (0.1 µg µL^−1^) + ds*SUC1* (0.1 µg µL^−1^) + ds*NUC1* (0.2 µg µL^−1^)

**Table 2:** Predicted species specificity of *Pseudococcus maritimus* dsRNA sequences, determined using si-Fi software. The *Planococcus* species are members of the same family (Pseudococcidae) of Coccoidea as *Ps. maritimus*, and *A. pisum* and *M. persicae* are aphids (superfamily Aphidoidea, sister taxon to superfamily Coccoidea).

dsRNA Sequence of *Ps. maritimus*	Insect Species	Number of Predicted Targets of dsRNA-Derived 21 nt Predicted Target Sequences
ds*AQP1*	*Planococcus citri*	27
	*Planococcus ficus*	17
	*Acyrthosiphon pisum*	0
	*Myzus persicae*	0
ds*SUC1*	*Planococcus citri*	0
	*Planococcus ficus*	0
	*Acyrthosiphon pisum*	0
	*Myzus persicae*	0
ds*NUC1*	*Planococcus citri*	9
	*Planococcus ficus*	1
	*Acyrthosiphon pisum*	0
	*Myzus persicae*	0

## Supplementary Materials

The following are available online at https://www.mdpi.com/2075-4450/11/11/739/s1, Figure S1. Rooted phylogenetic trees were constructed depicting relationship between AQP proteins using (A) Bayesian method (bootstrap values are shown on the branches) and (B) maximum likelihood method [the numbers on the branches are Shimodaira–Hasegawa approximate likelihood-ratio test (SH-aLRT) support (%)/ultrafast bootstrap support (%)]. Bacterial aquaporin proteins were used as an outgroup. Abbreviations: AQPZ, aquaporin Z; BIB, neurogenic protein big brain; DRIP, *Drosophila* integral protein family; EGLP, entomoglyceroporin; PRIP, *Pyrocoelia rufa* integral protein family. (C) Alignment of *AQP-1* gene of *Acyrthosiphon pisum* (accession number ACYPI006387), *Pseudococcus. maritimus* (accession number MT187985) and *Planococcus ficus* (accession number MT192030). *Ps. maritimus* dsRNA primer binding sites are highlighted yellow and qPCR primer binding sites are highlighted blue. Phylogenetic information was used to design degenerate primers for *AQP1*. The aquaporin sequences in Figure S1A,B are: Insects Sternorrhyncha (Hemiptera): aphids—*Aphis craccivora* [GAJW01004051.1], *A. craccivora* [KAF0764833.1], *A. curvipes* [GAJV01000737.1], *A. glycines* [KAE9531406.1], *A. glycines* [KAE9532649.1], *A. glycines* [KAE9534820.1], *A. gossypii* [AQZ36557.1], *A. gossypii* [XP027843520.1], *A. gossypii*[ XP027848076.1], *A. pisum* [ACYPI006387, *A. pisum* [ACYPI009194-PA], *A. pisum* [ACYPI010017], *Cinara cedri* [VVC32829.1], *C. cedri* [VVC43224.1], *Diuraphis noxia* [XP015366196.1], *D. noxia* [XP015367453.1], *Myzus ascalonicus* [FO032683.1], *M. ascalonicus* [GAAF01000345.1], *Macrosiphum euphorbiae* [GAOM01002305.1], *Myzus persicae* [XP022161341.1], *M. persicae* [XP022172799.1], *M. persicae* [XP022173314.1], *Melanaphis sacchari* [XP025197871.1], *M. sacchari* [XP025198278.1], *M. sacchari* [XP025200968.1], *Rhopalosiphum maidis* [XP026806330.1], *R. maidis* [XP026808959.1], *R. maidis* [XP026812299.1], *Rhopalosiphum padi* [AJL33751.1], *Sipha flava* [XP025405491.1], *S. flava* [XP025406216.1], *S. flava* [XP025409656.1]; whiteflies—*Bemisia tabaci* [APA28759.1], *B. tabaci* [APA28760.1], *B. tabaci* [APA28761.1], *B. tabaci* [APA28762.1], *B. tabaci* [XP018906592.1], *B. tabaci* [XP018908128.1], *B. tabaci* [XP018913501.1]; mealybugs—*Planococcus citri* [AQP-1, MT187985], *Pl. citri* [AQP-2], *Pl. citri* [AQP-3], *Pl. citri* [AQP-4], *Pl. citri* [AQP-5], *Pl. citri* [AQP-6], *Pl. citri* [AQP-7], *Pl. citri* [AQP-8], *Pl. ficus* [AQP-1, MT192030], *Ps maritimus* [AQP-1], *Phenacoccus solenopsis* [AYN64406.1]. psyllids—*Bactericera cockerelli* [AHB86600.1], *B. cockerelli* [AHB86601.2], *B. cockerelli* [AHB86602.2], *B. cockerelli* [AHB86603.1], *Diaphorina citri* [GACJ01000325.1], *D. citri* [GACJ01023397.1], *D. citri* [XP008484232.1], *D. citri* [XP008487274.1]; Auchenorrhyncha (Hemiptera): leafhoppers—*Cicadella viridis* [Q23808.1], *Homalodisca vitripennis* [DN195967.1]; planthoppers—Laodelphax striatellus [RZF36961.1], *Nilaparvata lugens* [XP022185065.1], *N. lugens* [XP022198592.1], *N. lugens* [XP022199860.1], *N. lugens* [XP022205785.1]. Heteroptera (Hemiptera):true bugs—*Apolygus lucorum* [KAE9438756.1], *Lygus hesperus* [AHI85743.1], *L. hesperus* [AHI85746.1], *L. hesperus* [AHI85749.1], *L. hesperus* [AHI85750.1], *Cimex lectularius* [AMZ04825.1], *C. lectularius* [AMZ04828.1], *C. lectularius* [AMZ04829.1], *C. lectularius* [AMZ04830.1], *Clavigralla tomentosicollisgi* [GAJX01000223.1], *C. tomentosicollisgi* [GAJX01005354.1], *Halyomorpha halys* [XP024216106.1]; *Rhodnius prolixus* [RPRC002014-PA], *R. prolixus* [RPRC002387-PA], *R. prolixus* [RPRC003319-PA], *R. prolixus* [RPRC003382-PA], *R. prolixus* [RPRC008707-PA], *R. prolixus* [RPRC011100-PA]; *Riptortus pedestris* [BAN21211.1]. Diptera: fruit flies—*Drosophila melanogaster* [NP611810.1, *EGLP1], D. melanogaster* [AAF58642.1.PRIP], *D. melanogaster* [AAM68740.2, *DRIP], D. melanogaster* [NP476837.1, *BIB], D. melanogaster* [NP611811.3, *EGLP2], D. melanogaster* [NP611812.2, EGLP3], *D. melanogaster* [NP611813.1, *EGLP4].* Bacteria—*Bacillus subtilis* [CUB53001.1-AQPZ], *B. subtilis* [WP015383197.1-Aquaglyceroporin], *Enterococcus faecalis* [STP66220.1-Aquaglyceroporin], *E. faecalis* [WP025188124.1-AQPZ], *Streptococcus pneumoniae* [CJR57869.1-Aquaglyceroporin], and *S. pneumoniae* [VTQ31887.1-AQPZ]. *Enterobacter cloacae* [WP129996330.1-AQPZ], *E. cloacae* [VAV80647.1-Aquaglyceroporin], *Escherichia. Coli* [AAC43518.1-AQPZ], *E. coli* [SYX52609.1-Aquaglyceroporin], *Pseudomonas aeruginosa* [SZD40238.1-Aquaglyceroporin], *P. aeruginosa* [WP029610146.1-AQPZ]. *A. pisum* and *R. prolixus* sequences were retrieved from aphidbase (https://bipaa.genouest.org/sp/acyrthosiphon_pisum/) and vectorbase (https://www.vectorbase.org/organisms/rhodnius-prolixus), respectively. The remaining sequences were downloaded from NCBI (https://blast.ncbi.nlm.nih.gov/Blast.cgi). Figure S2. Rooted phylogenetic trees were constructed depicting the relationship between sucrase proteins using (A) Bayesian method (bootstrap values are shown on the branches) and (B) maximum likelihood method (the numbers on the branches are SH-aLRT support (%)/ultrafast bootstrap support (%). *Drosophila melanogaster* sucrase proteins were used as an outgroup. (C) Alignment of *SUC-1* gene of *Acyrthosiphon pisum* (accession number ACYPI000002), *Pseudococcus maritimus* (accession number MT187986) and *Planococcus ficus* (accession number MT192031). *Ps. maritimus SUC1*-dsRNA primer binding sites are highlighted blue and qPCR primer binding sites are highlighted yellow. Phylogenetic information was used to design degenerate primers for *SUC1*. The sucrase sequences used in Figure S2A,B are: Insects Sternorrhyncha (Hemiptera): aphids—*Aphis craccivora* [KAF0746146.1], *A. craccivora* [KAF0766705.1], *A. craccivora* [KAF0767588.1], *A. glycines* [AG007859-PA], *A. glycines* [AG007860-PA], *A. glycines* [AG012057-PA], *A. glycines* [AG012058-PA], *A. glycines* [AG012059-PA], *A. glycines* [AG015450-PA], *A. glycines* [AG016935-PA], *A. glycines* [AG018074-PA], *A. glycines* [AG018961-PA], *A. gossypii* [XP_027847697.1], *A. gossypii* [XP_027847702.1], *A. pisum* [ACYPI000002-PA], *A. pisum* [ACYPI000768-PA], *A. pisum* [ACYPI002659-PA], *A. pisum* [ACYPI007753-PA], *A. pisum* [ACYPI008964-PA], *A. pisum* [XP_001952163.2], *A. pisum* [XP_003246887.1], *A. pisum* [XP_016659005.1], *Cinara cedri* [VVC30056.1], *C. cedri* [VVC30062.1], *C. cedri* [VVC30063.1], *Diuraphis noxia* [XP_015374664.1], *D. noxia* [XP_015375687.1], *D. noxia* [XP_015375756.1], *Myzus persicae* [XP_022164993.1], *M. persicae* [XP_022171053.1], *M. persicae* [XP_022171472.1], *M. persicae* [XP_022171483.1], *M. persicae* [XP_022171586.1], *M. persicae* [XP_022171691.1], *M. persicae* [XP_022171737.1], *M. persicae* [XP_022171816.1], *Melanaphis sacchari* [XP_025200270.1], *M. sacchari* [XP_025200431.1], *M. sacchari* [XP_025201971.1], *Rhopalosiphum maidis* [XP_026808057.1], *R. maidis* [XP_026808060.1], *R. maidis* [XP_026808063.1], *R. maidis* [XP_026808310.1], *R. maidis* [XP_026811817.1], *R. maidis* [XP_026811838.1], *R. maidis* [XP_026814773.1], *R. maidis* [XP_026821988.1], *Sipha flava* [XP_025406535.1], *S. flava* [XP_025411858.1], *S. flava* [XP_025411859.1], *S. flava* [XP_025411872.1], *S. flava* [XP_025411875.1], *S. flava* [XP_025411876.1], *S. flava* [XP_025411886.1], and *S. flava* [XP_025411887.1]*;* whiteflies—*Bemisia tabaci* [XP_018895643.1], *B. tabaci* [XP_018895757.1], *B. tabaci* [XP_018895821.1], *B. tabaci* [XP_018896838.1], *B. tabaci* [XP_018907630.1], *B. tabaci* [XP_018907649.1], *B. tabaci* [XP_018907799.1], *B. tabaci* [XP_018907832.1], *B. tabaci* [XP_018908637.1], *B. tabaci* [XP_018909206.1], *B. tabaci* [XP_018909322.1], *B. tabaci* [XP_018910471.1], *B. tabaci* [XP_018910472.1], *B. tabaci* [XP_018914092.1], *B. tabaci* [XP_018916040.1], *B. tabaci* [XP_018916662.1], *B. tabaci* [XP_018917258.1], *B. tabaci* [XP_018917350.1], *B. tabaci* [XP_018917351.1], *B. tabaci* [XP_018917500.1], *B. tabaci* [XP_018917587.1]; mealybugs—*Planococcus citri* [SUC1], *Pl. citri* [SUC4], *Pl. citri* [SUC5], *Pl. ficus* [SUC1, MT192031], *Ps. maritimus* [SUC1, MT187986], *Ps. maritimus* [SUC4, MT187987]. Auchenorrhyncha (Hemiptera): planthopper—*Laodelphax striatellus* [RZF35393.1], *L. striatellus* [RZF38899.1], *Nilaparvata lugens* [XP_022191426.1], *N. lugens* [XP_022191429.1], *N. lugens* [XP_022191432.1], *N. lugens* [XP_022191433.1], *N. lugens* [XP_022200264.1]. Diptera: fruit flies—*Drosophila melanogaster* [AAY55205.1], *D. melanogaster* [NP_001188791.2], *D. melanogaster* [NP_001286198.1], *D. melanogaster* [NP_476625.2], *D. melanogaster* [NP_476627.3], *D. melanogaster* [NP_476628.2], *D. melanogaster* [NP_609522.1], *D. melanogaster* [NP_610381.1], *D. melanogaster* [NP_610382.2], *D. melanogaster* [NP_610384.1], *D. melanogaster* [NP_724679.2]. *A. pisum* and *R. prolixus* sequences were retrieved from aphidbase (https://bipaa.genouest.org/sp/acyrthosiphon_pisum/) and vectorbase (https://www.vectorbase.org/organisms/rhodnius-prolixus), respectively. The remaining sequences were downloaded from NCBI (https://blast.ncbi.nlm.nih.gov/Blast.cgi). Figure S3. Rooted phylogenetic trees were constructed depicting relationship between DNA/RNA non-specific nuclease using (A) Bayesian method (bootstrap values are shown on the branches) and (B) maximum likelihood method (the numbers on the branches are SH-aLRT support (%)/ultrafast bootstrap support (%). Endonuclease G proteins from different species were used as an outgroup. (C) Alignment of *NUC1* gene of *Acyrthosiphon pisum* (accession number ACYPI008471), *Pseudococcus. maritimus* (accession number MT187988) and *Planococcus ficus* (accession number MT192032). *Ps. maritimus NUC1*-dsRNA primer binding sites are highlighted blue and qPCR primer binding sites are highlighted yellow. Phylogenetic information was used to design degenerate primers for *NUC1*. The sucrase sequences used in Figure S3A,B are: Insects Sternorrhyncha (Hemiptera): aphids—*A. pisum* [ACYPI008471], *Aphis craccivora* [KAF0769485.1], *A. craccivora* [KAF0771557.1], *A. glycines* [KAE9531558.1], *A. gossypii* [XP_027843039.1], *Cinara cedri* [VVC35734.1], *Diuraphis noxia* [XP_015375167.1], *Melanaphis sacchari* [XP_025194442.1]*, Myzus persicae* [XP_022183034.1], *Rhopalosiphum maidis* [XP_026811795.1]; *Sipha flava* [XP_025416859.1]; whiteflies—*Bemicia. Tabaci* [AQU43106.1], *B. tabaci* [AQU43107.1]; mealybugs—*Planococcus citri* [NUC1], *Pl. ficus* [NUC1, MT192032], *Ps. maritimus* [NUC1, MT187988]. Auchenorrhyncha (Hemiptera): Psyllids—*Diaphorina citri* [XP_008483858.1], *D. citri* [XP_015375305.1]; the planthopper—*Laodelphax* *striatellus* [QCB20006.1], *L. striatellus* [RZF42780.1], *Nilaparvata lugens* [XP_022200679.1]. Hymenoptera: honey bees*- Apis mellifera* [XP_624107.3]. Diptera: fruit flies—*Drosophila melanogaster* [NP_649076.1], *D. melanogaster* [NP_649078.1]. Coleoptera: beetles—*Tribolium castaneum* [XP_970494.1], *T. castaneum* [XP_973587.1]. Heteroptera: bugs—*Rhodnius prolixus* [RPRC015371]. Lepidoptera: moths—*Bombyx mori* [AK383943.1], *B. mori* [NP_001091744.1], *B. mori* [XP_004922835.1], *B. mori* [XP_004925361.1]. Orthoptera: locusts- *Schistocerca gregaria* [AHN55088.1], *S. gregaria* [AHN55089.1], *S. gregaria* [AHN55090.1], *S. gregaria* [AHN55091.1]. Nematoda: nematodes- *Brugia malayi* [XP_001892352.1], *Caenorhabditis elegans* [NP_491371.1], *Trichinella spiralis* [XP_003375679.1]. Mammalia: cow—*Bos Taurus* [CAA51320.1]; humans—*Homo sapiens* [AAX29001.1]; mouse—*Mus musculus* [NP_031957.1]. Crustacea: crab—*Chionoecetes opilio* [BAI79321.2], amphipod—*Gammarus_*sp. [ABI18973.1], shrimp—*Pandalus borealis* [CBG22733.1], *Penaeus monodon* [ABF69938.1]. Bacteria*—**Salmonella enterica* [AAL91099.1], *Serratia marcescens* [AAA26560.1]. Virus—white spot syndrome virus (WSSV) [AAW88443.1]. *A. pisum* and *R. prolixus* sequences were retrieved from aphidbase (https://bipaa.genouest.org/sp/acyrthosiphon_pisum/) and vectorbase (https://www.vectorbase.org/organisms/rhodnius-prolixus), respectively. The remaining sequences were downloaded from NCBI (https://blast.ncbi.nlm.nih.gov/Blast.cgi). Figure S4. Alignment of *βtub* gene of *Acyrthosiphon pisum* (accession number ACYPI001007) and *Pseudococcus. maritimus* (accession number MT187989). *Ps. maritimus βtub* qPCR primer binding sites are highlighted in yellow. Figure S5. The dsRNA target sequence alignment of *Pseudococcus maritimus* genes with the sequences of homologous genes from other species with predicted potential of non-target activity (A) *Ps maritimus* ds*AQP1* (250 bp) with *Planococcus citri AQP1*; (B) *Ps. maritimus* ds*AQP1* with *Pl. ficus AQP1*; (C) *Ps. maritimus* ds*NUC1* (250 bp) with *Pl. ficus NUC1*; (D) *Ps. maritimus* ds*NUC1* with *Pl*. *ficus NUC1*. Perfect sequence matches that equal or exceed 21 bp are highlighted in yellow. Text S1. Phylogenetic analysis of candidate genes. Table S1. Primer sequences. Table S2. PCR amplification conditions to amplify gene regions. Table S3. Statistical analysis of the effect of RNAi treatments on *P. maritimus* survival. Table S4 Statistical analysis of the effect of RNAi treatments on the expression of *P. maritimus* genes. Dataset S1. Mortality of *Pseudococcus maritimus* in RNAi experiments.
