# Supplementary material for: Evaluation of RNA Interference for Control of the Grape Mealybug Pseudococcus maritimus (Hemiptera: Pseudococcidae)
Source: Insects. 2020 Oct 28;11(11):739. doi: 10.3390/insects11110739 (PMC7692628; doi:10.3390/insects11110739)
Supplement: Supplementary file 1 [file insects-11-00739-s001.zip › supplementary/Supp_Fig_S1_AQP.pdf]

Fig. S1(A)

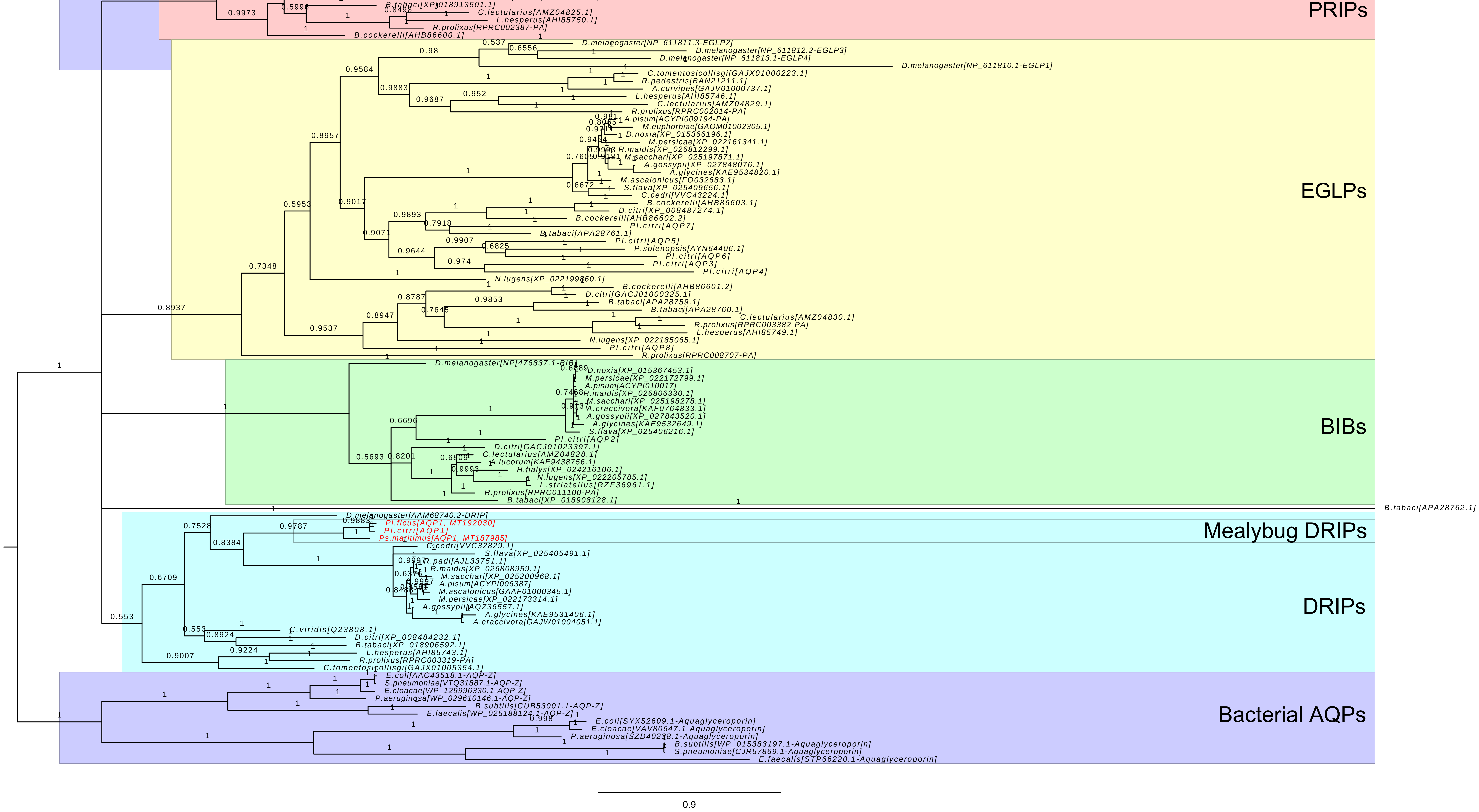

PRIPs
D.melanogaster[AAF58642.1-PRIP]
N.lugens[XP_022198592.1]
H.vitripennis[DN195967.1]
B.tabaci[XP_018913501.1]
C.lectularius[AMZ04825.1]
L.hesperus[AHI85750.1]
R.prolixus[RPRC002387-PA]
B.cockerelli[AHB86600.1]
EGLPs
D.melanogaster[NP_611811.3-EGLP2]
D.melanogaster[NP_611812.2-EGLP3]
D.melanogaster[NP_611813.1-EGLP4]
D.melanogaster[NP_611810.1-EGLP1]
C.tomentosicollisgi[GAJX01000223.1]
R.pedestris[BAN21211.1]
A.curvipes[GAJV01000737.1]
L.hesperus[AHI85746.1]
C.lectularius[AMZ04829.1]
R.prolixus[RPRC002014-PA]
A.pisum[ACYPI009194-PA]
M.euphorbiae[GAOM01002305.1]
D.noxia[XP_015366196.1]
M.persicae[XP_022161341.1]
R.maidis[XP_026812299.1]
M.sacchari[XP_025197871.1]
A.gossypii[XP_027848076.1]
A.glycines[KAE9534820.1]
M.ascalonicus[FO032683.1]
S.flava[XP_025409656.1]
C.cedri[VVC43224.1]
B.cockerelli[AHB86603.1]
D.citri[XP_008487274.1]
B.cockerelli[AHB86602.2]
Pl.citri[AQP7]
B.tabaci[APA28761.1]
Pl.citri[AQP5]
P.solenopsis[AYN64406.1]
Pl.citri[AQP6]
Pl.citri[AQP3]
Pl.citri[AQP4]
N.lugens[XP_022199860.1]
B.cockerelli[AHB86601.2]
D.citri[GACJ01000325.1]
B.tabaci[APA28759.1]
B.tabaci[APA28760.1]
C.lectularius[AMZ04830.1]
R.prolixus[RPRC003382-PA]
L.hesperus[AHI85749.1]
N.lugens[XP_022185065.1]
Pl.citri[AQP8]
R.prolixus[RPRC008707-PA]
BIBs
D.melanogaster[NP_476837.1-BIB]
D.noxia[XP_015367453.1]
M.persicae[XP_022172799.1]
A.pisum[ACYPI010017]
R.maidis[XP_026806330.1]
M.sacchari[XP_025198278.1]
A.craccivora[KAF0764833.1]
A.gossypii[XP_027843520.1]
A.glycines[KAE9532649.1]
S.flava[XP_025406216.1]
Pl.citri[AQP2]
D.citri[GACJ01023397.1]
C.lectularius[AMZ04828.1]
A.lucorum[KAE9438756.1]
H.halys[XP_024216106.1]
N.lugens[XP_022205785.1]
L.striatellus[RZF36961.1]
R.prolixus[RPRC011100-PA]
B.tabaci[XP_018908128.1]
B.tabaci[APA28762.1]
Mealybug DRIPs
D.melanogaster[AAM68740.2-DRIP]
Pl.ficus[AQP1, MT192030]
Pl.citri[AQP1]
Ps.maritimus[AQP1, MT187985]
DRIPs
C.cedri[VVC32829.1]
S.flava[XP_025405491.1]
R.padi[AJL33751.1]
R.maidis[XP_026808959.1]
M.sacchari[XP_025200968.1]
A.pisum[ACYPI006387]
M.ascalonicus[GAAF01000345.1]
M.persicae[XP_022173314.1]
A.gossypii[AQZ36557.1]
A.glycines[KAE9531406.1]
A.craccivora[GAJW01004051.1]
C.viridis[Q23808.1]
D.citri[XP_008484232.1]
B.tabaci[XP_018906592.1]
L.hesperus[AHI85743.1]
R.prolixus[RPRC003319-PA]
C.tomentosicollisgi[GAJX01005354.1]
Bacterial AQPs
E.coli[AAC43518.1-AQP-Z]
S.pneumoniae[VTQ31887.1-AQP-Z]
E.cloacae[WP_129996330.1-AQP-Z]
P.aeruginosa[WP_029610146.1-AQP-Z]
B.subtilis[CUB53001.1-AQP-Z]
E.faecalis[WP_025188124.1-AQP-Z]
E.coli[SYX52609.1-Aquaglyceroporin]
E.cloacae[VAV80647.1-Aquaglyceroporin]
P.aeruginosa[SZD40238.1-Aquaglyceroporin]
B.subtilis[WP_015383197.1-Aquaglyceroporin]
S.pneumoniae[CJR57869.1-Aquaglyceroporin]
E.faecalis[STP66220.1-Aquaglyceroporin]
0.9

Fig. S1(B)

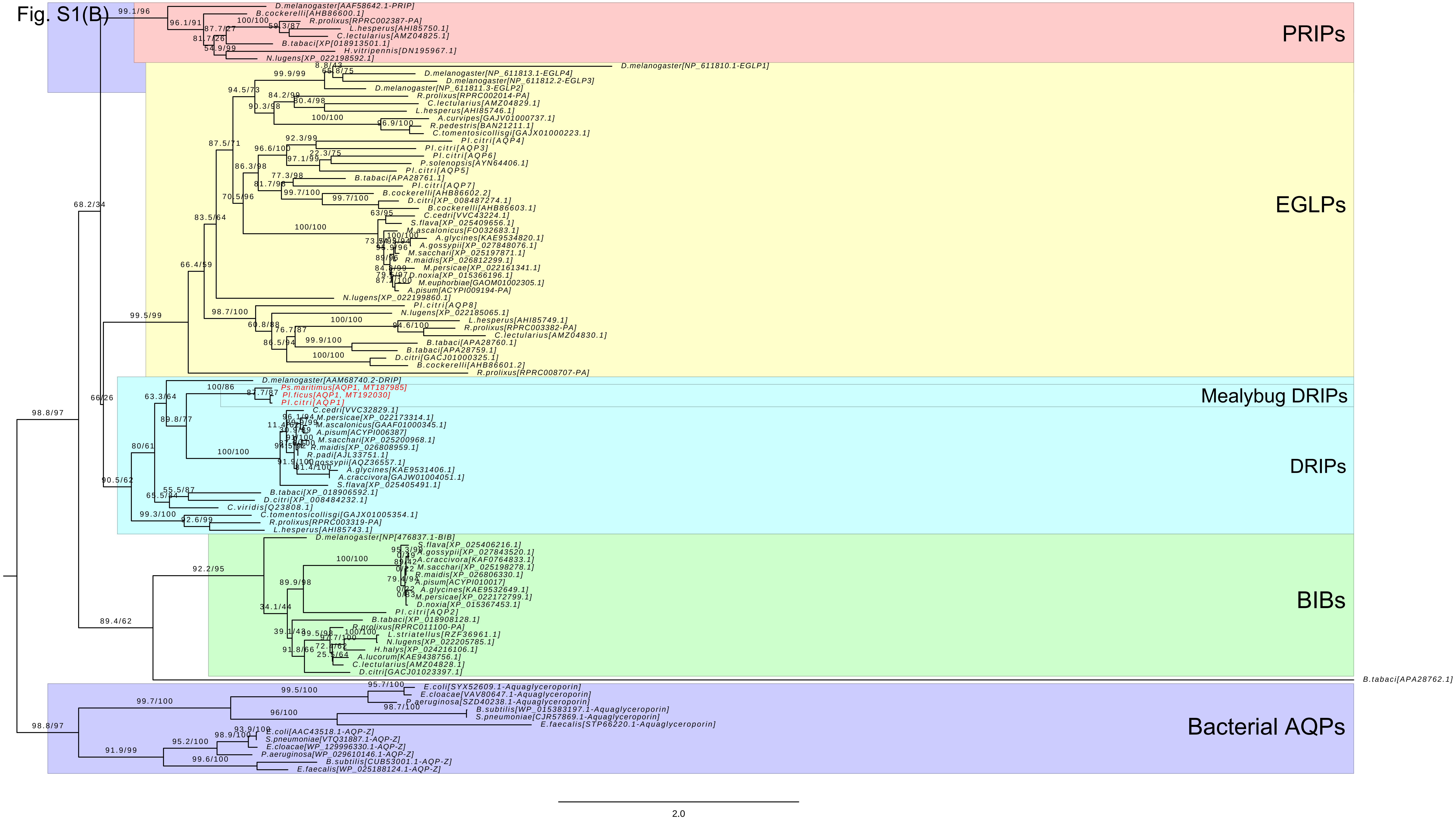
PRIPs
D.melanogaster[AAF58642.1-PRIP]
B.cockerelli[AHB86600.1]
R.prolixus[RPRC002387-PA]
L.hesperus[AHI85750.1]
C.lectularius[AMZ04825.1]
B.tabaci[XP_018913501.1]
H.vitripennis[DN195967.1]
N.lugens[XP_022198592.1]
EGLPs
D.melanogaster[NP_611810.1-EGLP1]
D.melanogaster[NP_611813.1-EGLP4]
D.melanogaster[NP_611812.2-EGLP3]
D.melanogaster[NP_611811.3-EGLP2]
R.prolixus[RPRC002014-PA]
C.lectularius[AMZ04829.1]
L.hesperus[AHI85746.1]
A.curvipes[GAJV01000737.1]
R.pedestris[BAN21211.1]
C.tomentosicollisgi[GAJX01000223.1]
Pl.citri[AQP4]
Pl.citri[AQP3]
Pl.citri[AQP6]
P.solenopsis[AYN64406.1]
Pl.citri[AQP5]
B.tabaci[APA28761.1]
Pl.citri[AQP7]
B.cockerelli[AHB86602.2]
D.citri[XP_008487274.1]
B.cockerelli[AHB86603.1]
C.cedri[VVC43224.1]
S.flava[XP_025409656.1]
M.ascalonicus[FO032683.1]
A.glycines[KAE9534820.1]
A.gossypii[XP_027848076.1]
M.sacchari[XP_025197871.1]
R.maidis[XP_026812299.1]
M.persicae[XP_022161341.1]
D.noxia[XP_015366196.1]
M.euphorbiae[GAOM01002305.1]
A.pisum[ACYPI009194-PA]
N.lugens[XP_022199860.1]
Pl.citri[AQP8]
N.lugens[XP_022185065.1]
L.hesperus[AHI85749.1]
R.prolixus[RPRC003382-PA]
C.lectularius[AMZ04830.1]
B.tabaci[APA28760.1]
B.tabaci[APA28759.1]
D.citri[GACJ01000325.1]
B.cockerelli[AHB86601.2]
R.prolixus[RPRC008707-PA]
Mealybug DRIPs
D.melanogaster[AAM68740.2-DRIP]
Ps.maritimus[AQP1, MT187985]
Pl.ficus[AQP1, MT192030]
Pl.citri[AQP1]
DRIPs
C.cedri[VVC32829.1]
M.persicae[XP_022173314.1]
M.ascalonicus[GAAF01000345.1]
A.pisum[ACYPI006387]
M.sacchari[XP_025200968.1]
R.maidis[XP_026808959.1]
R.padi[AJL33751.1]
A.gossypii[AQZ36557.1]
A.glycines[KAE9531406.1]
A.craccivora[GAJW01004051.1]
S.flava[XP_025405491.1]
B.tabaci[XP_018906592.1]
D.citri[XP_008484232.1]
C.viridis[Q23808.1]
C.tomentosicollisgi[GAJX01005354.1]
R.prolixus[RPRC003319-PA]
L.hesperus[AHI85743.1]
BIBs
D.melanogaster[NP[476837.1-BIB]
S.flava[XP_025406216.1]
A.gossypii[XP_027843520.1]
A.craccivora[KAF0764833.1]
M.sacchari[XP_025198278.1]
R.maidis[XP_026806330.1]
A.pisum[ACYPI010017]
A.glycines[KAE9532649.1]
M.persicae[XP_022172799.1]
D.noxia[XP_015367453.1]
Pl.citri[AQP2]
B.tabaci[XP_018908128.1]
R.prolixus[RPRC011100-PA]
L.striatellus[RZF36961.1]
N.lugens[XP_022205785.1]
H.halys[XP_024216106.1]
A.lucorum[KAE9438756.1]
C.lectularius[AMZ04828.1]
D.citri[GACJ01023397.1]
B.tabaci[APA28762.1]
Bacterial AQPs
E.coli[SYX52609.1-Aquaglyceroporin]
E.cloacae[VAV80647.1-Aquaglyceroporin]
P.aeruginosa[SZD40238.1-Aquaglyceroporin]
B.subtilis[WP_015383197.1-Aquaglyceroporin]
S.pneumoniae[CJR57869.1-Aquaglyceroporin]
E.faecalis[STP66220.1-Aquaglyceroporin]
E.coli[AAC43518.1-AQP-Z]
S.pneumoniae[VTQ31887.1-AQP-Z]
E.cloacae[WP_129996330.1-AQP-Z]
P.aeruginosa[WP_029610146.1-AQP-Z]
B.subtilis[CUB53001.1-AQP-Z]
E.faecalis[WP_025188124.1-AQP-Z]
99.1/96
96.1/91
100/100
59.3/87
87.7/27
81.7/26
54.9/99
68.2/34
99.9/99
94.5/73
84.2/99
90.3/98
100/100
96.9/100
92.3/99
87.5/71
96.6/100
97.1/99
86.3/98
77.3/98
81.7/96
99.7/100
99.7/100
70.5/96
83.5/64
100/100
63/95
66.4/59
99.5/99
98.7/100
100/100
60.8/88
76.7/87
86.5/94
99.9/100
100/100
66/26
63.3/64
100/86
89.8/77
80/61
100/100
90.5/62
55.5/87
65.5/84
99.3/100
92.6/99
98.8/97
92.2/95
100/100
89.9/98
34.1/44
89.4/62
39.1/43
91.8/66
99.7/100
99.5/100
95.7/100
96/100
98.7/100
98.8/97
93.9/100
98.9/100
95.2/100
91.9/99
99.6/100
2.0

Fig. S1(C)

```
A.pisum_AQP1      TTTTTGGGCACGGCTATTCTATTGTTTCTTGGTTGTGGCAGCATTATGTGGCTTAACGGT
P.maritimus_AQP1  -------------------TGCTGTTCCTCGGCTGCGGTACCATTATGAACGTCGAAGGC
P.ficus_AQP1      -------------------TGCTTTTCTTCGGCTGCGGTACCATTATGAACGTTAACAAA
                                     *..* **..*.**.**.**.* ******* .  *..* ..

A.pisum_AQP1      TCAACTAATTCATCAGATATCCTAGCAATCTCTCTGACTTTTGGTTTCACAATAGCAACA
P.maritimus_AQP1  ------GCCGTGCTGGTAGTTCAAGTTGCCT---TAACTTTCGGCCTGACTATCGCCGTG
P.ficus_AQP1      ------GACATTTTAACCGTTCAAGTAGCCT---TAACTTTTGGCCTATGTATCGCAGTT
                        . . . ....  .*.* **. ..**   *.*****.**..*    ** ** ..

A.pisum_AQP1      CTAGTACAGATATTCGGTCAGACCAGTGGATGTCATATCAACCCAGCAGTCACGGTTAGC
P.maritimus_AQP1  TTAGCCCAAAGTATCGGCCATGTGAGTGGATGTCACATAAATCCTGCGGTTACGCTGAGT
P.ficus_AQP1      ATGGCACAGAGTATCGGCCATGTGAGTGGATGTCACATAAATCCTGCGGTTACGCTCAGT
                   *.*. **.*   ****.** .. ***********.** **.** **.**.*** * **.

A.pisum_AQP1      TTTTTGGTGAGCGGCCAATGCTCCTTTTTGAAATCTGCTCTGTACATAGCTGCACAGTGT
P.maritimus_AQP1  TTTCTGGTTGTTGGTAAATGCTCGATTCTGAAATCGTTGTGTTACATTGTTTTACAATGC
P.ficus_AQP1      TTTCTGATTGTTGGTAAATGCTCGATTCTGAAATCGTTATGTTACATTGTTTTACAATGC
                  ***.**.* . .**. *******  **.*******  . .  ***** *.* .***.**.

A.pisum_AQP1      TTGGGGGCTATAGCTGGTATCTACCTTCTAGAATTTGTTACTCCAGACG---------CA
P.maritimus_AQP1  GCTGGCGCTACAGCTGGGTTTTATGTTTTAACTCTTCTGACCCCACAAGGCATTCTAACA
P.ficus_AQP1      GCTGGAGCTGCAGCTGGGTTTTATGTTTTACTTCTCGTGACCCCTCATGCTACCGAAGCT
                   . ** ***..******  *.**. **.**   .*. * **.**  * *         *

A.pisum_AQP1      GTAACTAAAGGCTTAGGAAAGACAGACATAAACACACTGCTTCAACCGGGACAAGGATTC
P.maritimus_AQP1  GCTAATAAAAACCTCGGAAATACATTCTTAGGACCGAAAGTTACACCTGTACAAGGGCTC
P.ficus_AQP1      TCGTCCAAAAACCTCGGAAATACATCTTTAGGAATTAATGTCACACCTTCACAAGGATTG
                   .   .***..*.* ***** ***  . **..  .     *.  ***   ******..*

A.pisum_AQP1      GTCGTAGAAGCATTCATTACCTTTATTCTAGTGTTGGTTATTCACTCGGTCTGCGATGAA
P.maritimus_AQP1  GGGATCGAGATTATTGCCACGTTTTTATTATGCTTCGTCATTCATTCAGTCTGCGATGAA
P.ficus_AQP1      GTAGTCGAGATCATCGCAACATTTTTATTATGCTTCGTAATTCATTCGGTTTGCGATGAA
                  *  .* **...  *... ** *** * .**   ** ** *****.**.**.*********
```

```
A.pisum_AQP1      GCCAACCGAAGCAACATAGTTACGCCGTCCATTTCCATAGGTCTGACCATTGCTGCCGCC
P.maritimus_AQP1  AGAAGAACAGATGTGAAAATGATTGCTCCTCTGTTGATCGGAATTTCAGCCGTTGTTTGC
P.ficus_AQP1      AGGAGATCAGATGTGAAAATGATTGCTCCTTTATTGATTGGAATTTCTGCCGTCGTTTGC
                  .  *.   *....  * *.* *.  * .*. * *. ** **  *  * ...*..*..  *

A.pisum_AQP1      CACCTAGCAGCGATTAAATACACAGGAGCTAGCATGAATCCAGCAAGATCTTTGGGTCCA
P.maritimus_AQP1  CATCTATTCGCTATCAAATTCACCGGTTCGAGTTTGAACCCAGCTCGT------------
P.ficus_AQP1      CATTTATTCGCAATTGATTACACTGGTTCGAGTTTGAATCCTGCTCGTAGTTTTGGCC--
                  **..** . ** **..* * *** **  * **. ****.** **  *

A.pisum_AQP1      GCCGTGGCTCTAGGTTCATGGTCAAACCATTGGGTATACTGGGTCGGTCCTATTGTCGGT
P.maritimus_AQP1  ------------------------------------------------------------
P.ficus_AQP1      ------------------------------------------------------------

A.pisum_AQP1      GGCATACTTGGAGGAACCGTCCACACTTTTGTGTTGAAACGTCATACTGAAGAGGCAAGC
P.maritimus_AQP1  ------------------------------------------------------------
P.ficus_AQP1      ------------------------------------------------------------

A.pisum_AQP1      TCTTATGATCTTTGA
P.maritimus_AQP1  ---------------
P.ficus_AQP1      ---------------
```
