# Supplementary material for: Evaluation of RNA Interference for Control of the Grape Mealybug Pseudococcus maritimus (Hemiptera: Pseudococcidae)
Source: Insects. 2020 Oct 28;11(11):739. doi: 10.3390/insects11110739 (PMC7692628; doi:10.3390/insects11110739)
Supplement: Supplementary file 1 [file insects-11-00739-s001.zip › supplementary/Supp_Fig_S2_SUC.pdf]

Fig. S2(A)

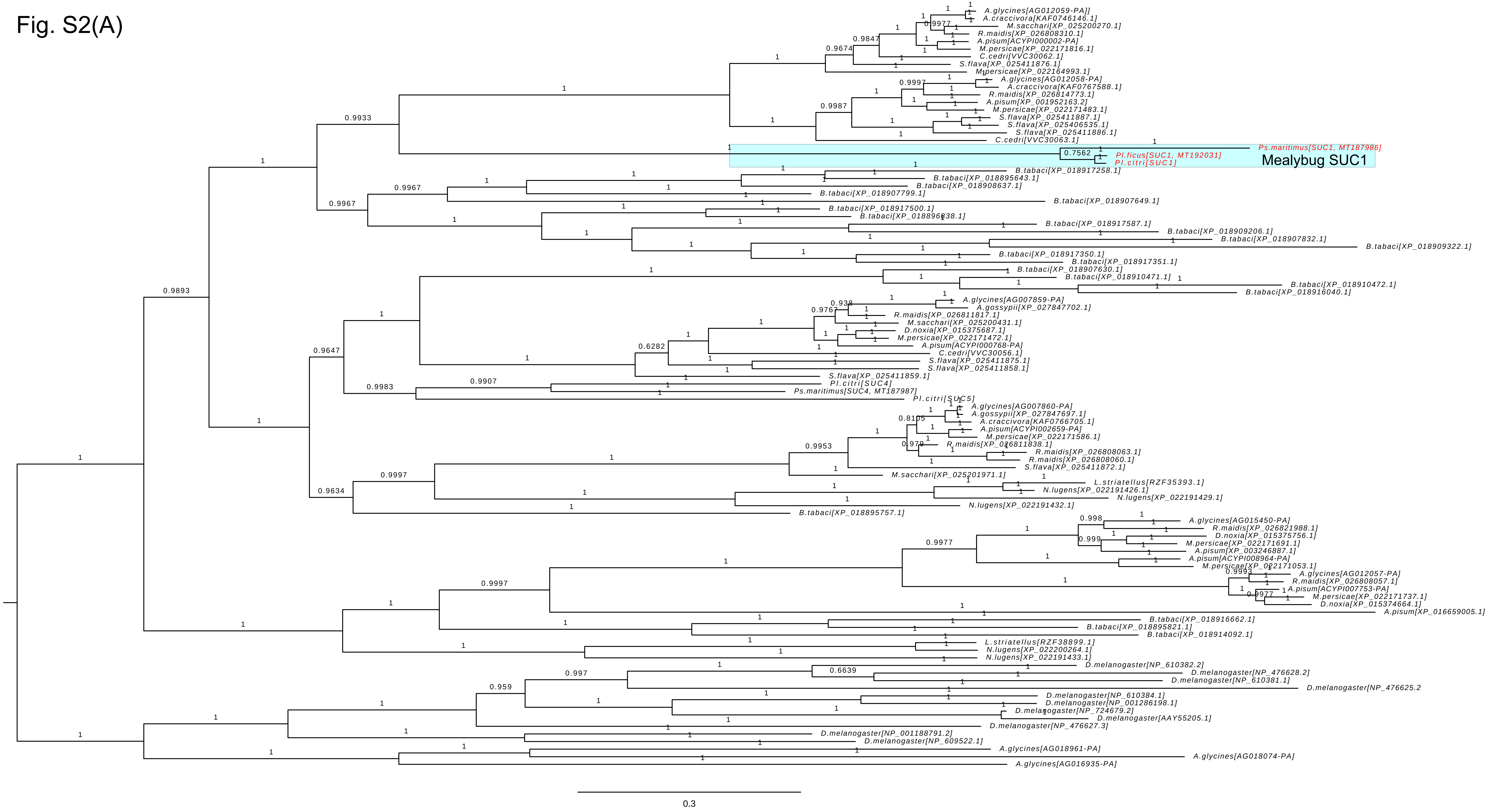
A.glycines[AG012059-PA]
A.craccivora[KAF0746146.1]
M.sacchari[XP_025200270.1]
R.maidis[XP_026808310.1]
A.pisum[ACYPI000002-PA]
M.persicae[XP_022171816.1]
C.cedri[VVC30062.1]
S.flava[XP_025411876.1]
M.persicae[XP_022164993.1]
A.glycines[AG012058-PA]
A.craccivora[KAF0767588.1]
R.maidis[XP_026814773.1]
A.pisum[XP_001952163.2]
M.persicae[XP_022171483.1]
S.flava[XP_025411887.1]
S.flava[XP_025406535.1]
S.flava[XP_025411886.1]
C.cedri[VVC30063.1]
Ps.maritimus[SUC1, MT187986]
Pl.ficus[SUC1, MT192031]
Pl.citri[SUC1]
Mealybug SUC1
B.tabaci[XP_018917258.1]
B.tabaci[XP_018895643.1]
B.tabaci[XP_018908637.1]
B.tabaci[XP_018907799.1]
B.tabaci[XP_018907649.1]
B.tabaci[XP_018917500.1]
B.tabaci[XP_018896838.1]
B.tabaci[XP_018917587.1]
B.tabaci[XP_018909206.1]
B.tabaci[XP_018907832.1]
B.tabaci[XP_018909322.1]
B.tabaci[XP_018917350.1]
B.tabaci[XP_018917351.1]
B.tabaci[XP_018907630.1]
B.tabaci[XP_018910471.1]
B.tabaci[XP_018910472.1]
B.tabaci[XP_018916040.1]
A.glycines[AG007859-PA]
A.gossypii[XP_027847702.1]
R.maidis[XP_026811817.1]
M.sacchari[XP_025200431.1]
D.noxia[XP_015375687.1]
M.persicae[XP_022171472.1]
A.pisum[ACYPI000768-PA]
C.cedri[VVC30056.1]
S.flava[XP_025411875.1]
S.flava[XP_025411858.1]
S.flava[XP_025411859.1]
Pl.citri[SUC4]
Ps.maritimus[SUC4, MT187987]
Pl.citri[SUC5]
A.glycines[AG007860-PA]
A.gossypii[XP_027847697.1]
A.craccivora[KAF0766705.1]
A.pisum[ACYPI002659-PA]
M.persicae[XP_022171586.1]
R.maidis[XP_026811838.1]
R.maidis[XP_026808063.1]
R.maidis[XP_026808060.1]
S.flava[XP_025411872.1]
M.sacchari[XP_025201971.1]
L.striatellus[RZF35393.1]
N.lugens[XP_022191426.1]
N.lugens[XP_022191429.1]
N.lugens[XP_022191432.1]
B.tabaci[XP_018895757.1]
A.glycines[AG015450-PA]
R.maidis[XP_026821988.1]
D.noxia[XP_015375756.1]
M.persicae[XP_022171691.1]
A.pisum[XP_003246887.1]
A.pisum[ACYPI008964-PA]
M.persicae[XP_022171053.1]
A.glycines[AG012057-PA]
R.maidis[XP_026808057.1]
A.pisum[ACYPI007753-PA]
M.persicae[XP_022171737.1]
D.noxia[XP_015374664.1]
A.pisum[XP_016659005.1]
B.tabaci[XP_018916662.1]
B.tabaci[XP_018895821.1]
B.tabaci[XP_018914092.1]
L.striatellus[RZF38899.1]
N.lugens[XP_022200264.1]
N.lugens[XP_022191433.1]
D.melanogaster[NP_610382.2]
D.melanogaster[NP_476628.2]
D.melanogaster[NP_610381.1]
D.melanogaster[NP_476625.2
D.melanogaster[NP_610384.1]
D.melanogaster[NP_001286198.1]
D.melanogaster[NP_724679.2]
D.melanogaster[AAY55205.1]
D.melanogaster[NP_476627.3]
D.melanogaster[NP_001188791.2]
D.melanogaster[NP_609522.1]
A.glycines[AG018961-PA]
A.glycines[AG018074-PA]
A.glycines[AG016935-PA]
0.3

Fig. S2(B)

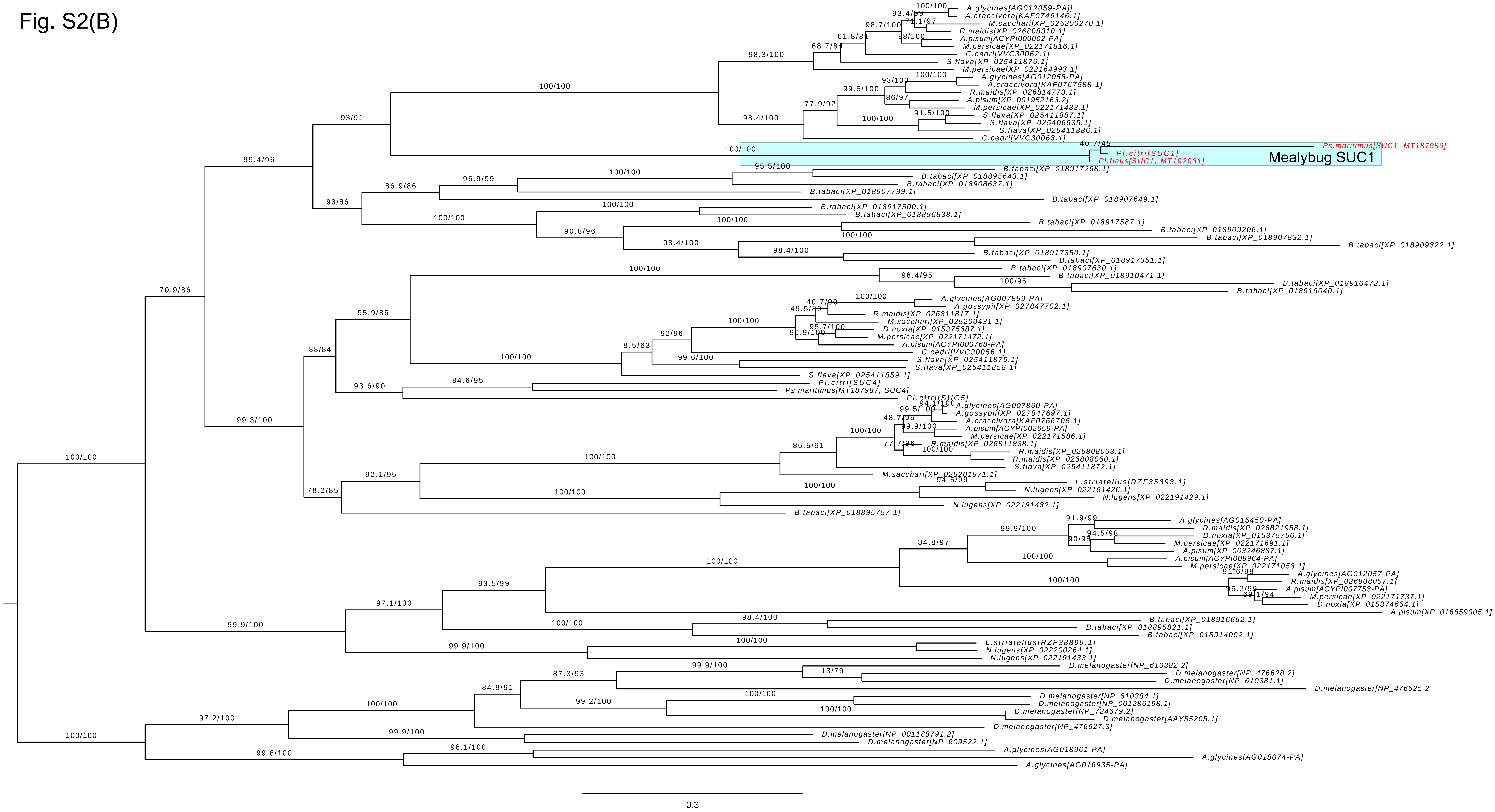
Mealybug SUC1
Ps.maritimus[SUC1, MT187986]
Pl.citri[SUC1]
Pl.ficus[SUC1, MT192031]
0.3

Fig. S2(C)

```
A.pisum_SUC1       ATGTTTAAGGTAATCACTGTGTGCATATGGCTATTCGCCTTCAACAGTCTTAATGTGTCG
P.maritimus_SUC1   ------------------------------------------------------------
P.ficus_SUC1       ------------------------------------------------------------

A.pisum_SUC1       TCCGAGTATGTTTATGAGGGTTTGAAGAGCGATTCGGTCGAACCAGACTGGTGGCAGACG
P.maritimus_SUC1   ------------------------------------------------------------
P.ficus_SUC1       ------------------------------------------------------------

A.pisum_SUC1       GAAATTATCTATCAAATATACGTAAGATCGTTTAAAGACAGCGATGGCGACGGAATCGGA
P.maritimus_SUC1   ------------------------------------------------------------
P.ficus_SUC1       ------------------------------------------------------------

A.pisum_SUC1       GACCTGAACGGTATAACAGAGAAAGTCCCATATTTTAAAACTATAGATGTTGGCGCCGTT
P.maritimus_SUC1   ------------------------------------------------------------
P.ficus_SUC1       ------------------------------------------------------------

A.pisum_SUC1       TGGTTGTCGCCAATATTCCTCTCACCACAAAATGATTTCGGATACGATATATCAGACTAC
P.maritimus_SUC1   ------------------------------------------------------------
P.ficus_SUC1       ------------------------------------------------------------

A.pisum_SUC1       AAAGAGATCGATCCTATTTATGGCTCAATGGCAGACTTTGAACGGATGAGGGATGAGTTT
P.maritimus_SUC1   ------------------------------------------------------------
P.ficus_SUC1       ------------------------------------------------------------

A.pisum_SUC1       CACAAGCATGGCATAAAGGTCTTATTGGACTTTGTGCCAAATCACACGAGTGACGAACAC
P.maritimus_SUC1   ------------------------------------------------------------
P.ficus_SUC1       ------------------------------------------------------------

A.pisum_SUC1       GAATGGTTCCAAAAGTCAATTAAGAAAATAGAGCCCTTTTCAGACTATTACGTATGGAAA
P.maritimus_SUC1   ------------------------------------------------------------
P.ficus_SUC1       ------------------------------------------------------------


A.pisum_SUC1       GATCCGGTCCGTGATGTACATGGAAATAACACGCCTCCGAGTAATTGGTTAGGTGTGTTC
P.maritimus_SUC1   ------------------------------------------------------------
P.ficus_SUC1       ------------------------------------------------------------
```

```
A.pisum_SUC1     AACAGTGGGTCTGCGTGGGAATGGAATGAAGAACGTCAACAATATTATTTACATCAGTTC
P.maritimus_SUC1 ------------------------------------------------------------
P.ficus_SUC1     ------------------------------------------------------------

A.pisum_SUC1     CAAGTGAAACAACCCGACTTGAACTACAGAAATCCGTCGGTT-AGAGAAGAAATAAAGAA
P.maritimus_SUC1 ----------------------------------TGGTTGTGCTACGAAGAAATGAAGAA
P.ficus_SUC1     -----------ACCAGACATCAATTTCAGAAATGCAGTTGTG-TACGAAGAAATGAAGAA
                                                   .. . **   . ********.*****

A.pisum_SUC1     CACGCTGTT--ATATTGGTTGGGACGTGGCGTCGACGGGTTCAGATTC-GACGCGGTGAA
P.maritimus_SUC1 TAATATTATACTTTTGGCTTATCCAAGGGTATCGATGGATTGCGAATCGGATTCGGCGAA
P.ficus_SUC1     TAACATCAT--TTTTTGGCTGTCAAAAGGAATCGATGGACTAAGAATT-GATTCAGCGAA
                 .*   *  *   * * * .*.    . ** .****.**..*  ** *. **. *.*.***

A.pisum_SUC1     CTATCTATTCGAAAGAGAAGATCTAGCTGACGAACCTAAGTCTAATAAAATTGGCTATTT
P.maritimus_SUC1 TTTCTTTATAGAAGATGAACGATTTTTGGATGAACCACTTTCAGGAGATTCTTTCGCATT
P.ficus_SUC1     TTTTCTAATTGAAGACGAACAATTTCTAGATGAACCACCATCAGGAGACACTTTTGCGTT
                 .* ..*  * ***.. *** . .*  . **.*****    ** .. .*  .*  .   **

A.pisum_SUC1     GGACACCGATTACGATTCTTTAACGCATACGAGCACACTTGATCAACCTGAAACTTATTC
P.maritimus_SUC1 GCCTGATGAGTACCCTTCGTTGAAACACATTTACACTCTAGACCGTCCGGAAAATGTTGA
P.ficus_SUC1     ACCTGATGAATATCTTTCGTTGAAACATCCTTATACTCTCGATAGACCTGAAAATATTGA
                 .  .. .** **.  *** **.* .**. .  ..** ** **. . ** **** *  *

A.pisum_SUC1     CATCGTTCGTCAATGGAGGCAGATGCTGGACAGTTACAGGACTAGGGAAAAGAAAACCAA
P.maritimus_SUC1 GATAATAAAAGACTGGAGGAAAATCTTCGATGAATATTCGAC---ACCACGACGACCCAA
P.ficus_SUC1     AATTATAAAAGACTGGAGAAAAATCTTTGATCAATATTCAAC---GAAACAAAAACCCAA
                  ** .*  .  * *****. *.** .* **. . **.  .**   .  * .. .* ****

A.pisum_SUC1     GTTTATGATGGTGGAATGTTACTCACCATTTGATAAAACTTTGTTGTACTACGGTAGTAA
P.maritimus_SUC1 AGTTATGATCACAGAAGCATATTCAAATGTGAAAAATATCCTTCCATTTTACGGAACGAG
P.ficus_SUC1     AATTATGATAACCGAAGCTTATTCAAATGTTAAGAACATCGTACCCTTTTATGGAACAGA
                 . ******* .. ***   **.***    * .* ** *.. * .. * .**.** *  ..

A.pisum_SUC1     TTCAGAGCCTGGCGCTCATTTTCCATTTAACTTTTTATTCATCGGAACGTTCGATCAACA
P.maritimus_SUC1 TGCAGAGCCCGGAGCTCATTTACCTTTTAATTTCTTAATGATTACTGAAGTAGGAAGGGA
P.ficus_SUC1     AGCAGAGCCCGGAGCACATTTACCGTTTAATTTTCTAATGATTACCGAAGTGGGCAGAGA
                   *******.** ** ***** ** *****.**..** * **..  . . * *.  .. *
```

```
A.pisum_SUC1      GTCTGATGCTGCTAAGGTCCATAACATGATCAAATCATGGATTCGTGGTATGCCCACCGG
P.maritimus_SUC1  ATCGAATGCTA-------------------------------------------------
P.ficus_SUC1      ATCAAACGCTC--AA---------------------------------------------
                  .** .*.***

A.pisum_SUC1      CATGTGGCCTAACTGGGTGTTAGGTAACCATGATAACGCAAGAGTGGCTTCGAGGAGCAA
P.maritimus_SUC1  ------------------------------------------------------------
P.ficus_SUC1      ------------------------------------------------------------

A.pisum_SUC1      TCCAATGTTAGTTGATGGACTACACATGATCCAACATCTGTTGCCCGGCACCTCTGTGAC
P.maritimus_SUC1  ------------------------------------------------------------
P.ficus_SUC1      ------------------------------------------------------------

A.pisum_SUC1      TTATTACGGAGACGAACTAGGTCTGATCGACACAACCGTTCGTTGGGACCAAACAGTTGA
P.maritimus_SUC1  ------------------------------------------------------------
P.ficus_SUC1      ------------------------------------------------------------

A.pisum_SUC1      TCCAGCGGGGCTTAACGTGGGCCCCTATAGGTTCTTGAAATTCAGCAGAGATCCCGTGAG
P.maritimus_SUC1  ------------------------------------------------------------
P.ficus_SUC1      ------------------------------------------------------------

A.pisum_SUC1      GACTCCGTTCCCATGGGACAGTTCGTATAACGCAGGTTTTTCTAATTCGTCTTCGTTGTG
P.maritimus_SUC1  ------------------------------------------------------------
P.ficus_SUC1      ------------------------------------------------------------

A.pisum_SUC1      GCTTCCTCTTAACGCCGATTATTGGAAAAAAAATATGGTTGAAGAATCAAGGTTTAAAAG
P.maritimus_SUC1  ------------------------------------------------------------
P.ficus_SUC1      ------------------------------------------------------------

A.pisum_SUC1      TAACCTAAGGTCATACAGGCAATTGGCTCGGTTAAGGAGGAGTCTCACATTTGTCAAAGG
P.maritimus_SUC1  ------------------------------------------------------------
P.ficus_SUC1      ------------------------------------------------------------

A.pisum_SUC1      CGATTTGCATTTATACACACTGTCCAAATGGGTGTTTGGATTTTCACGGAGTTTTTATGA
P.maritimus_SUC1  ------------------------------------------------------------
P.ficus_SUC1      ------------------------------------------------------------
```

```
A.pisum_SUC1      TCACCCGACTTACTTCATTGTAGTTAATTTCGGGAGTGAAATAGAAACAGTTAACTTGAT
P.maritimus_SUC1 ------------------------------------------------------------
P.ficus_SUC1      ------------------------------------------------------------

A.pisum_SUC1      GGAAGCTAGAGGTACTTTACCATTAACTATGAAAGTTAAGGTATCTAGCATTAACTCTGG
P.maritimus_SUC1 ------------------------------------------------------------
P.ficus_SUC1      ------------------------------------------------------------

A.pisum_SUC1      TTTTGTTACCGGAAATTTAGTACGTACTGATAGTGTATTACTGCGCCCGAAAGCAGCTCT
P.maritimus_SUC1 ------------------------------------------------------------
P.ficus_SUC1      ------------------------------------------------------------

A.pisum_SUC1      TGTACTTACAACCTCAAGAATGAACGAAGATACATAA
P.maritimus_SUC1 -------------------------------------
P.ficus_SUC1      -------------------------------------
```
