# Supplementary material for: Evaluation of RNA Interference for Control of the Grape Mealybug Pseudococcus maritimus (Hemiptera: Pseudococcidae)
Source: Insects. 2020 Oct 28;11(11):739. doi: 10.3390/insects11110739 (PMC7692628; doi:10.3390/insects11110739)
Supplement: Supplementary file 1 [file insects-11-00739-s001.zip › supplementary/Supp_Fig_S3_NUC.pdf]

Fig. S3(A)

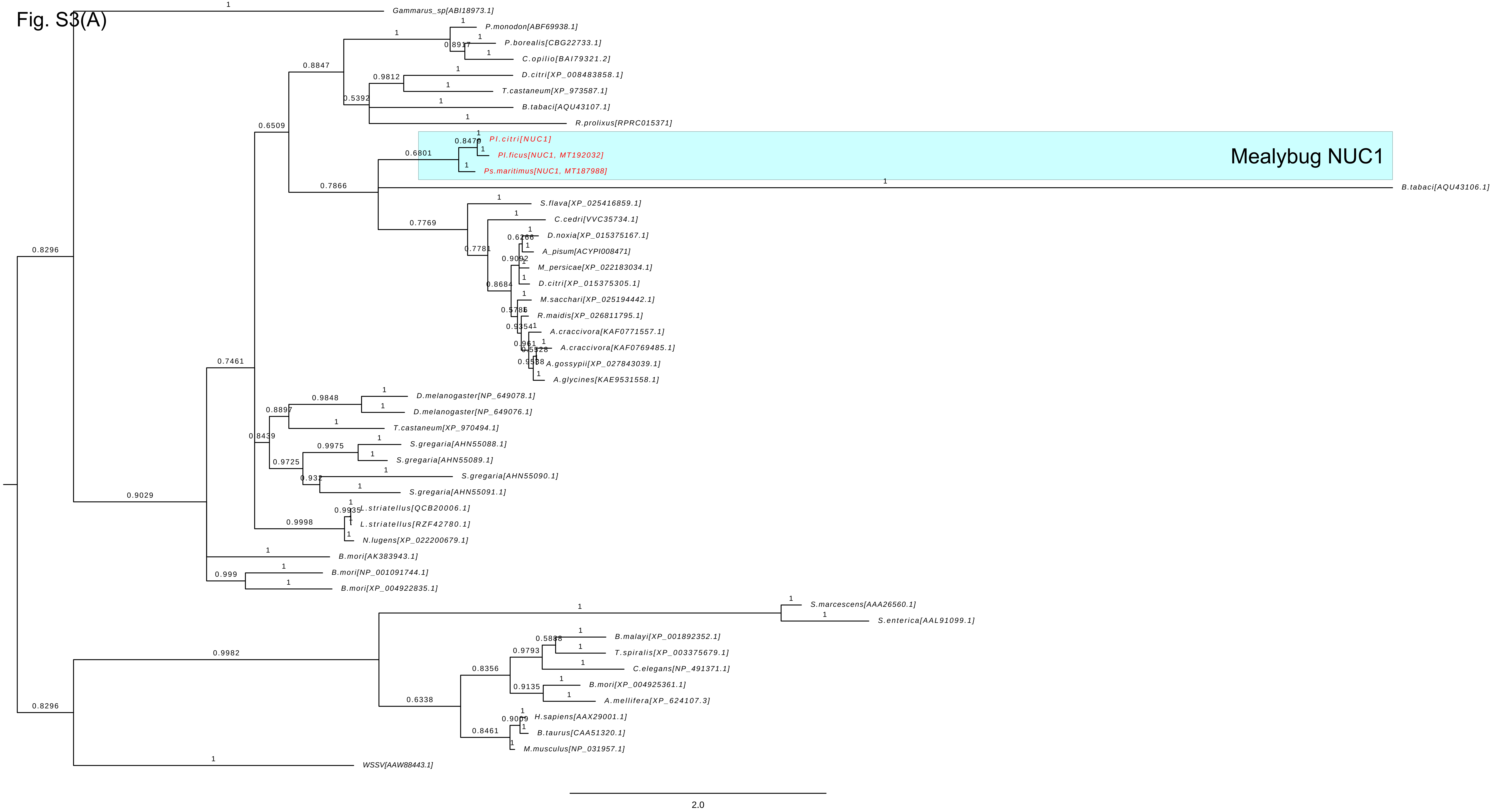
Gammarus_sp[ABI18973.1]
P.monodon[ABF69938.1]
P.borealis[CBG22733.1]
C.opilio[BAI79321.2]
D.citri[XP_008483858.1]
T.castaneum[XP_973587.1]
B.tabaci[AQU43107.1]
R.prolixus[RPRC015371]
Pl.citri[NUC1]
Pl.ficus[NUC1, MT192032]
Ps.maritimus[NUC1, MT187988]
Mealybug NUC1
B.tabaci[AQU43106.1]
S.flava[XP_025416859.1]
C.cedri[VVC35734.1]
D.noxia[XP_015375167.1]
A_pisum[ACYPI008471]
M_persicae[XP_022183034.1]
D.citri[XP_015375305.1]
M.sacchari[XP_025194442.1]
R.maidis[XP_026811795.1]
A.craccivora[KAF0771557.1]
A.craccivora[KAF0769485.1]
A.gossypii[XP_027843039.1]
A.glycines[KAE9531558.1]
D.melanogaster[NP_649078.1]
D.melanogaster[NP_649076.1]
T.castaneum[XP_970494.1]
S.gregaria[AHN55088.1]
S.gregaria[AHN55089.1]
S.gregaria[AHN55090.1]
S.gregaria[AHN55091.1]
L.striatellus[QCB20006.1]
L.striatellus[RZF42780.1]
N.lugens[XP_022200679.1]
B.mori[AK383943.1]
B.mori[NP_001091744.1]
B.mori[XP_004922835.1]
S.marcescens[AAA26560.1]
S.enterica[AAL91099.1]
B.malayi[XP_001892352.1]
T.spiralis[XP_003375679.1]
C.elegans[NP_491371.1]
B.mori[XP_004925361.1]
A.mellifera[XP_624107.3]
H.sapiens[AAX29001.1]
B.taurus[CAA51320.1]
M.musculus[NP_031957.1]
WSSV[AAW88443.1]
2.0

Fig. S3(B)

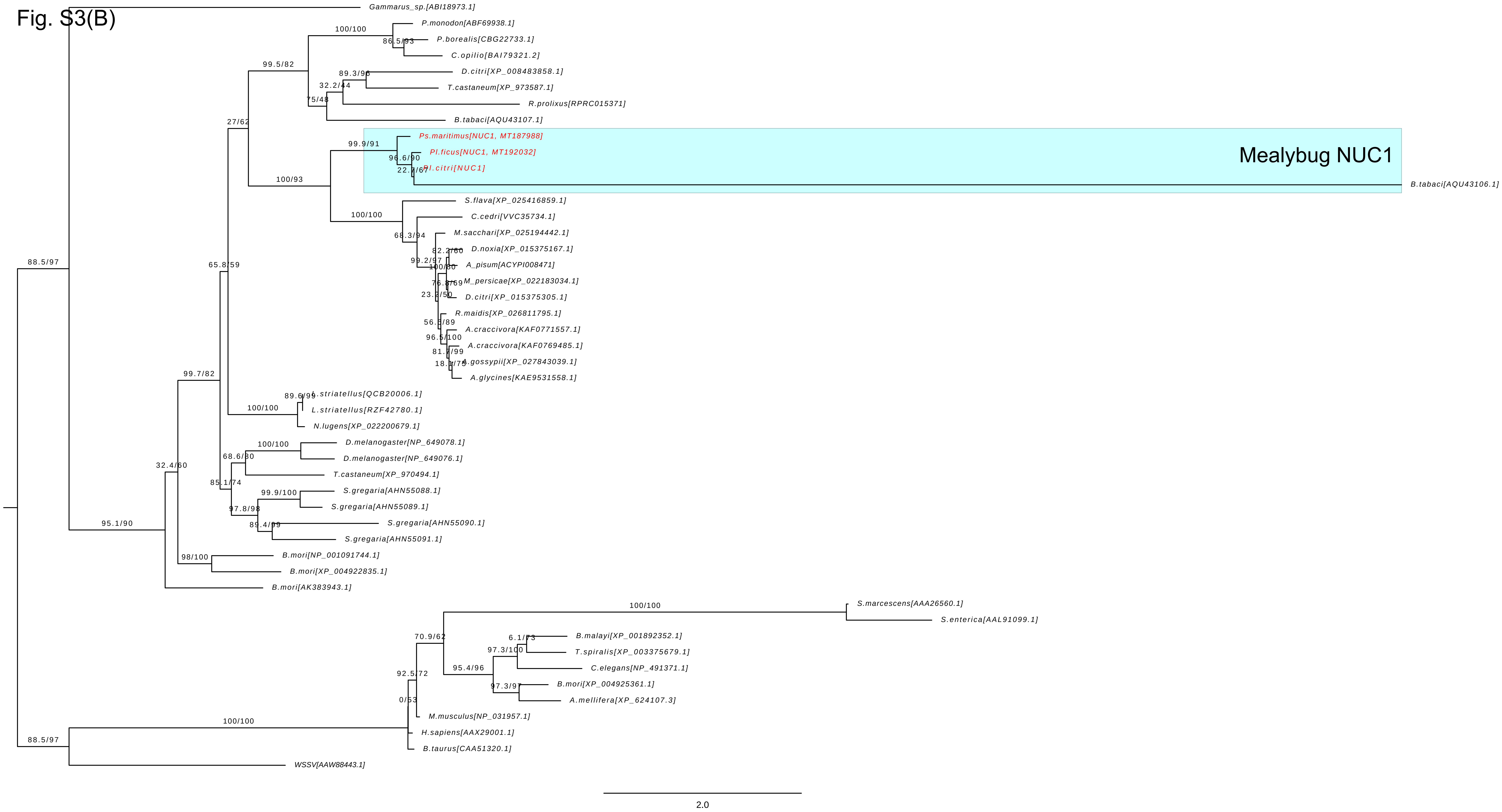

Gammarus_sp.[ABI18973.1]
P.monodon[ABF69938.1]
P.borealis[CBG22733.1]
C.opilio[BAI79321.2]
D.citri[XP_008483858.1]
T.castaneum[XP_973587.1]
R.prolixus[RPRC015371]
B.tabaci[AQU43107.1]
Ps.maritimus[NUC1, MT187988]
Pl.ficus[NUC1, MT192032]
Pl.citri[NUC1]
B.tabaci[AQU43106.1]
Mealybug NUC1
S.flava[XP_025416859.1]
C.cedri[VVC35734.1]
M.sacchari[XP_025194442.1]
D.noxia[XP_015375167.1]
A_pisum[ACYPI008471]
M_persicae[XP_022183034.1]
D.citri[XP_015375305.1]
R.maidis[XP_026811795.1]
A.craccivora[KAF0771557.1]
A.craccivora[KAF0769485.1]
A.gossypii[XP_027843039.1]
A.glycines[KAE9531558.1]
L.striatellus[QCB20006.1]
L.striatellus[RZF42780.1]
N.lugens[XP_022200679.1]
D.melanogaster[NP_649078.1]
D.melanogaster[NP_649076.1]
T.castaneum[XP_970494.1]
S.gregaria[AHN55088.1]
S.gregaria[AHN55089.1]
S.gregaria[AHN55090.1]
S.gregaria[AHN55091.1]
B.mori[NP_001091744.1]
B.mori[XP_004922835.1]
B.mori[AK383943.1]
S.marcescens[AAA26560.1]
S.enterica[AAL91099.1]
B.malayi[XP_001892352.1]
T.spiralis[XP_003375679.1]
C.elegans[NP_491371.1]
B.mori[XP_004925361.1]
A.mellifera[XP_624107.3]
M.musculus[NP_031957.1]
H.sapiens[AAX29001.1]
B.taurus[CAA51320.1]
WSSV[AAW88443.1]
100/100
86.5/93
89.3/96
32.2/44
75/48
99.5/82
27/62
99.9/91
96.6/90
22.7/67
100/93
100/100
68.3/94
99.2/97
82.2/60
100/80
76.8/69
23.2/50
56.5/89
96.5/100
81.7/99
18.1/75
65.8/59
88.5/97
99.7/82
89.6/99
100/100
100/100
68.6/30
85.1/74
99.9/100
97.8/98
89.4/99
32.4/60
95.1/90
98/100
100/100
70.9/62
6.1/73
97.3/100
95.4/96
97.3/97
92.5/72
0/53
100/100
88.5/97
2.0

Fig. S3(C)

```
A.pisum_NUC1      ATGCATTCAACTGCATTAATTACTGCAGTATGTTTAGTCATTTGTTCGTGTCTTCTTCCG
P.maritimus_NUC1  ------------------------------------------------GTTTTCTC---
P.ficus_NUC1      A-----------------------------------------ATGCGAGTGCTTCT---
                                                                 ** .*...

A.pisum_NUC1      ACCATCAATGCCAAGAAAATCAATTATCAAAATGTTAAAAACGAGGACGACTGTTCGTTA
P.maritimus_NUC1  ------------------------------------------------------------
P.ficus_NUC1      ------------------------------------------------------------

A.pisum_NUC1      TGGGTGGCCGAAAAAGACGAGCCCAAAATGCCCATACCGTTCACGAGCAACGGCAAGCGG
P.maritimus_NUC1  -------ATTAAACGATCATGTACCAAAAACCATCATC-TTGATGAGTGCAACCCAAAAT
P.ficus_NUC1      -------CATTAACGAACACGTTCCAAAAACCGCCATC-ATAACCAGCATCACTCAACAA
                            ** .. *. *. * *** .**  .*.*  * *. **..  . . *. .

A.pisum_NUC1      TACGCGATCATCTATCCGAACGCCAAGGGCCGGCTGGACGTGAAGAAAGGGCGTAGTTTC
P.maritimus_NUC1  AACCAAATGCTCTACCCGAGCGAATCCAATACAATCAGAATGCGTACCGGGACCCGGTTC
P.ficus_NUC1      AACCAAATACTTTATCCGAACACAACCAATACGCTCAAATTAGCCACTGGAGGCAGGATA
                  **  .**  *.**.****.*.     ...  . * ..  *.   *  **.  . *  *

A.pisum_NUC1      AAGCTGTCGTGCGCCACAGCCAAGTTCGCGTCGAGCGAGCTGCAACGGAACGGCACCTCC
P.maritimus_NUC1  AAGGTAAGCTGCGGCGATAAAGACTTCAAGAAAAAATTCAAAAAATCACCAAGAACCAAA
P.ficus_NUC1      AGGCTAACCTGCGGTGACAAATACTTCAAGAAAAAATTTAAAAAATCGTCAAAAACCAAG
                *.* *.   **** ..  .   * ***. *  .*.      . **. .   .. ***

A.pisum_NUC1      GAAGTGTTGGTCACGTGCGTCGGCATCGACGTCCTCGCGTACCGGGGCCAGACGTACCGG
P.maritimus_NUC1  GAAGTCCAAGCACGTTGCAATTCCAAAGACATAATCAATGTCGAAGGCGAACGAATACGT
P.ficus_NUC1      GAGGTTGTGGCACGTTGCAATTCCAAAGACGTCGTCAACGTTGAAAATGAACGAATACGA
                **.**   .*.    ***. .  **  ***.*  **.    . ..... *.  .   **

A.pisum_NUC1      TACGAAGACTTCCAGTGCGACGGCATGCCCAAGTCGGAGCTCCGAGTCACCGACGATCGG
P.maritimus_NUC1  TTTCGAGAACTAGAATGTCAAAGTTTTCCAACATCCAAACCCCAAAAAAGAGAAAATAAA
P.ficus_NUC1      CTTCGAGAACTAGAATGTGAAGATTTCCCATCATCAAAGCCGCACAAGAAGACCGAAAAG
                . . .*** .*  *.**. * ... * **   .** .*.*. *. .  *  .  .*  ..
A.pisum_NUC1      TGCCAGCCGGCCAACTACACCGTGGCCGTGGTCGGTTTCCAGACGGACCACGCATTCCTC
P.maritimus_NUC1  AAATGCCATGGAAACAACACGCTCTTCGATATAGGATTTCCAACCCGCGACAATTTTTTG
P.ficus_NUC1      AAATGCCATCAAAACAATACCTTGTTTGATATCGCTTTTGAAGTTCGTGGTGGTTCTTTG
                  . .. *     *** *.**  *  ..*  .* *  **.  ...  .. ...  *...*

A.pisum_NUC1      CGGCTGTACGGCATGTGTTTCGACAAGTCGACCAAGAACAGCCTGTACACTTGGTACGAC
P.maritimus_NUC1  GATATGATTCGAGTATGTTTCGATGAACTACAACAGGAGTCTCGTTACACCTGGTATGAT
P.ficus_NUC1      GACTCGATACGAGCATGTTTTGACGAACCGAATCAAGATTCGATTTACACGTGGTACGAT
                  .  .*    * ...*****.**..*....    *..*       ***** *****.**.
```

```
A.pisum_NUC1     GCCCGGGCGCCGTACTACGACAACCACCAGAAGTACAGCAAGCGACCGGCGTTCATTAAG
P.maritimus_NUC1 TC---GTCGATGCTACCAACAGGACATCAGAGTAACGTAGGAAGACCTCGATTCGTCCA-
P.ficus_NUC1     AC---GTCGATGTTACCCACAGGACATCAGAGTAACGTCAAAAGACCCCAATTCGCGCA-
                 *   * ** .*.  .  .  .. **.****. **.  ... ****   .***..  *

A.pisum_NUC1     TCCAAAGAGCTGTACGGCAACACGGACGTGAACAGGAAATACACTTTCAAAGAACAGAAA
P.maritimus_NUC1 --CGATAATCTCTACAGGTTCCCGGTCG---ACGAGGTGTACAAGAGTAGTTACCAGCAC
P.ficus_NUC1     --CGATGATTTGTATAGATTTCTTGTCT---ACGAAGCATATACCATTAATCATCAACGT
                  *.* .* .* **..*   . . * *    **.... .**.*    .*.  * **. .

A.pisum_NUC1     AAAACGGTGGCGAAAATACTCAGATCGGACGAACTTGCGGATAAGTACATAAGGAATGAT
P.maritimus_NUC1 GATTGGTTTACCAAATTGTTAAAATCTCGAGAAAAAGCTGATCAATATATCAAAAACGAC
P.ficus_NUC1     GATCAGTTTGCTAATTTGTTACAATCGAAAGAAAAGGCTGATGAATATATTAAAAACGAT
                .*   * * .* **  *..*  .***  . ***   ** *** *.**.** *..**.**.

A.pisum_NUC1     AACCAACACTCGCTGTCCCGGGGCCATTATGCAGCCAAAGCTGACTTCTTCTTTGATTTC
P.maritimus_NUC1 GGCGAACATTTTCTGTCCAGAGGGCATCTTACGCCAAAGGCTGATATGGTTTATGGATCG
P.ficus_NUC1     GGCAAGCATTTTTTGTCCAGAGGTCATCTCACAGCAAAAGCTGACATGGTGTATGGATCG
                ..* *.**.*. .***** *.** ***. ..*. * **.*****. *  * * **. *.

A.pisum_NUC1     GAACAGATTTCAACGTTTTACTATGCGAACGTAGCCCCACAGTGGCAGATATTCAACGGT
P.maritimus_NUC1 GAACAATCAGCAACTTTCCATTATATCAACGTGGCACCTCAGTGGCAAGGTTTCAACGGT
P.ficus_NUC1     GAGCAGTCGGCTACTTTTCATTATATCAATGTCGCACCTCAATGGCAATGTTTTAATGAT
                **.**. .  * ** **..*.***.. **.** ** ** **.*****.   **.**.*.*

A.pisum_NUC1     GACATGTGGGCCGATCTGGAGCAGTCAACCCGGTCAAAGTTAAGCAAAGGAAACGGCACG
P.maritimus_NUC1 GGTAATTGGAATAAAGTTGAACAAAGTGCAAGAGAAGAGCTCGAGAAAAAGGATAA----
P.ficus_NUC1     GGTAATTGGAATAAAGTAGAAGATAGCGTCAGGAAAGAGATCCAGAAGAAGGATAA----
                *..*  ***. ..*  * **. *    ..  *.  *.** *  . **.....*...

A.pisum_NUC1     TCCACACATGTGATCGTCACCGGTACGTACGACACATGCACACTGGCCGACGTGGACAAC
P.maritimus_NUC1 --AAGATACCGTGTGGTAACCGGAACCTACGGAGTGGCCACGCTACCAGACGTAAACAAC
P.ficus_NUC1     --AAGATACCGAGTCGTAACTGGAACCCACGGAATAGCCACACTACCCGACGTAAACAAC
                   * *.*.   .* ** **.** ** .***. ...  ***.**. * *****..*****

A.pisum_NUC1     GTCCAACAACCACTGTACCTTGACCTGCCTGGTGCCATACGCGTGCCGCT----------
P.maritimus_NUC1 AACGAACAAGAACTCTACCTCTACGAAGACGAGAACAAAAACCCATTGCTCA--------
P.ficus_NUC1     AACGAGCAAGAACTCTACCTTTACGAGGACGAGAACAAGAAACCCTTACTCAAAGTGCCT
                . * *.***  *** *****. **  .  .*. . ** . .  . ...**
```

```
A.pisum_NUC1      --GTTCTACTGGAAGCTGCACTACGATGTGGACGCGGCGGACGGCATCGTGTACATCGGC
P.maritimus_NUC1 ------------------------------------------------------------
P.ficus_NUC1      AAGCTTTTTTGGAAACTAGTCTACGACCTGATCG--------------------------

A.pisum_NUC1      CTCAACAACCCGTACAAGGAGATCGACGACAGCGTGTACATATGTCCTAACATATGCCCG
P.maritimus_NUC1 ------------------------------------------------------------
P.ficus_NUC1      ------------------------------------------------------------

A.pisum_NUC1      GACGGTTACCATGGTCGCGGTTACCAGGGCCGCGATCACCGAAACAATAAGGCAGAGACC
P.maritimus_NUC1 ------------------------------------------------------------
P.ficus_NUC1      ------------------------------------------------------------

A.pisum_NUC1      GGACCCGGCCGCGATCACCGGAACGACCCCGAACCGGACGCCAACGACGGACTCATCTAC
P.maritimus_NUC1 ------------------------------------------------------------
P.ficus_NUC1      ------------------------------------------------------------

A.pisum_NUC1      TGCTGTACCAAAAAGTCGTTCGAAGAGGTTTACGGTGAGCTGGACCCAATCGTGTACAGA
P.maritimus_NUC1 ------------------------------------------------------------
P.ficus_NUC1      ------------------------------------------------------------

A.pisum_NUC1      CAGCTGATG
P.maritimus_NUC1 ---------
P.ficus_NUC1      --------G
```
