# Supplementary material for: Evaluation of RNA Interference for Control of the Grape Mealybug Pseudococcus maritimus (Hemiptera: Pseudococcidae)
Source: Insects. 2020 Oct 28;11(11):739. doi: 10.3390/insects11110739 (PMC7692628; doi:10.3390/insects11110739)
Supplement: Supplementary file 1 [file insects-11-00739-s001.zip › supplementary/Supp_Fig_S4_Btub.pdf]

Fig. S4

```
A.pisum_βtub      ATGAGGGAAATCGTACACTTGCAAGCCGGACAATGCGGAAACCAGATCGGCGCCAAGTTC
P.maritimus_βtub  --------------------------------------------------AGCTAAATTT
                                                                     **.**.**.

A.pisum_βtub      TGGCAAGTCATATCCGAAGAGCACGGAATCGACTATAC-AGGATTCTACAAGGGCACATC
P.maritimus_βtub  TGGGAAATTATCTCCGATGAACCGGGTATCGACGCTACTGGAAACTTATCACGGAGATTC
                  *** **.*.** ***** **.*  ** ******  *** .*.* ..**. * ** .  **

A.pisum_βtub      CGATCTACAGCTGGAACGCATTAACGTTTACTATAACGAAGGGTCTGCCGCCAACCGTTC
P.maritimus_βtub  CGATTTACAATTAGAAAGAATTAATGTGTACTACAATGAAGCCTCT--------------
                  ****.****..*.*** * *****.** *****.**.****  ***

A.pisum_βtub      GGAAGGTGGAAAATATGTGCCACGAGCCGTCTTACTCGACCTGGAACCCGGTACCATGGA
P.maritimus_βtub  ----GGCGGTAAATATGTACCTCGTGCTATCTTGGTCGATTTGGAACCCGGTACCATGGA
                      **.** ********.** ** **..****. ***..*******************

A.pisum_βtub      GTCCATCCGGTCCGGACCTTACGGCGCGCTTTTCCGGCCGGACAACTTCGTGTTCGGTCA
P.maritimus_βtub  TTCCGTTCGTGCCGGACCTTTCGGTCAAATTTTCCGACCTGATAACTTCGTATTCGGCCA
                   ***.*.**  ********* ***.  . *******.****.********.*****.**

A.pisum_βtub      GTCGGGCGCTGGAAACAACTGGGCCAAGGGTCATTACACTGAGGGTGCCGAACTCGTGGA
P.maritimus_βtub  GTCTGGCGCTGGTAACAATTGGGCCAAAGGACACTACACCGAAGGTGCCGAATTAGTCGA
                  **  ******** *****.********.** **.*****.**.*********.* ** **

A.pisum_βtub      CGCCGTGTTGGACGTGGTTCGCAAAGAGAGCGAGAACTGTGACTGTTTGCAGGGATTCCA
P.maritimus_βtub  CTCAGTTTTGGATGTTGTCAGGAAAGAAGCCGAAAGTTGCGATTGCTTACAAGGTTTTCA
                  * * ** *****.** **. * *****.. ***.*..**.**.**.**.**.** **.**

A.pisum_βtub      GTTGGCGCACTCGTTGGGCGGTGGCACCGGTTCCGGACTGGGTACACTTCTGATATCGAA
P.maritimus_βtub  ATTGACTCACTCGTTGGGAGGCGGTACCGGTTCCGGTATGGGTACCTTACTTATTTCTAA
                  .***.* *********** **.**.***********  ******* .* ** ** ** **

A.pisum_βtub      GATCCGCGAGGAGTACCCTGACCGCATAATGAATACGTACTCAGTGATGCCATCTCCAAA
P.maritimus_βtub  AATACGTGAAGAATATCCCGATAGAATAATGAACACTTACTCTGTTGTACCATCGCCCAA
                  .** **.**.**.**.**.**. * ********.** ***** ** .*.***** ** **

A.pisum_βtub      GGTGTCGGACA-CGGTGGTGGAGCCGTACAACGCGACCTTGTCCGTGCACCAGCTGGTGG
P.maritimus_βtub  AGTATCTGACACCAGTTGTTGAACCTTACAATGCCACATTATCAGTCCACCAATTGGTTG
                   .**.** **** *.** ** **.** *****.** ** **.** ** *****..****
*

A.pisum_βtub      AGAATACCGACG-AGTCGTACCTG-ATCGACAACGAGGCGCTGTACGACATTTGCTTCCG
P.maritimus_βtub  AAAACACCGACGAAACCATATCTGTATCGATAACGAAGCCcTATACGA-ATCTGCTTCAG
                  *.**.******* *..*.**.*** *****.*****.**  *.***** **.****** *

A.pisum_βtub      GACGCTGAAGCTGACCAACCCGACGTACGGCGACCTCAACCACCTTGTGTCGCTGACAAT
P.maritimus_βtub  AACGTTGAAACTCACCACC-----------------------------------------
                  .***.****.** **** *

A.pisum_βtub      GTCCGGCGTGACCACGTGCCTCCGGTTCCCCGGTCAGCTGAACGCCGACCTCCGCAAGCT
P.maritimus_βtub  ------------------------------------------------------------

A.pisum_βtub      GGCGGTCAACATGGTCCCGTTCCCCAGGCTCCACTTCTTCATGCCGGGATTCGCGCCTCT
P.maritimus_βtub  ------------------------------------------------------------
```

```
A.pisum_βtub       TACGGCCCGCGGAAGTCAGTCTTACCGGGCCATGAGCGTGCCCGAGCTCACTCAGCAAAT
P.maritimus_βtub   ------------------------------------------------------------

A.pisum_βtub       GTTCGACGCCAAGAACATGATGGTCGCCTGCGACCCACGACACGGCCGCTACCTCACCGT
P.maritimus_βtub   ------------------------------------------------------------

A.pisum_βtub       CGCCGCCATATTCAGGGGTCGCATGTCCATGAAAGAGGTGGACGAGCAGATGTTGAACGT
P.maritimus_βtub   ------------------------------------------------------------

A.pisum_βtub       GCAAAACAAGAACTCGAGCTACTTTGTCGATTGGATCCCAAACAACGTGAAAACGGCCGT
P.maritimus_βtub   ------------------------------------------------------------

A.pisum_βtub       TTGCGATATCCCGCCATGCGGTCTCAAGATGAGCTCCACGTTCATCGGAAACACCACGGC
P.maritimus_βtub   ------------------------------------------------------------

A.pisum_βtub       CATCCAAGAGCTGTTCAAGCGCATATCCGAACAGTTTTCGGCCATGTTCAGAAGGAAGGC
P.maritimus_βtub   ------------------------------------------------------------

A.pisum_βtub       GTTCTTGCATTGGTACACCGGCGAAGGTATGGATGAGATGGAATTCACCGAAGCTGAATC
P.maritimus_βtub   ------------------------------------------------------------

A.pisum_βtub       CAACATGAACGATTTGATCTCCGAATACCAACAGTATCAGGAGGCATCGGTGGACGAGGA
P.maritimus_βtub   ------------------------------------------------------------

A.pisum_βtub       GTACATAGAAGAAGAAGAAACCGAAGAGACAGATATGTGCGAT
P.maritimus_βtub   -------------------------------------------
```
