# Supplementary material for: Evaluation of RNA Interference for Control of the Grape Mealybug Pseudococcus maritimus (Hemiptera: Pseudococcidae)
Source: Insects. 2020 Oct 28;11(11):739. doi: 10.3390/insects11110739 (PMC7692628; doi:10.3390/insects11110739)
Supplement: Supplementary file 1 [file insects-11-00739-s001.zip › supplementary/Supp_Fig_S5_dsRNA.pdf]

Fig. S5(A)

```
P.maritimus_dsAQP1         ------------------------------------------------------------
P.citri_AQP1               ATGACTGGTTACCAAGGGCCGCAAAAACCGAAAGAAAATATGGGTAATGACATGAAAATC

P.maritimus_dsAQP1         ------------------------------------------------------------
P.citri_AQP1               TTAGGAGTAGAAGAATTCACCGAAAGCAATAAAATTTGGCGCATGCTATGCGCCGAATTT

P.maritimus_dsAQP1         ------------------------------------------------------------
P.citri_AQP1               CTGGGCGCGTTCTCATTGCTTTTCTTCGGCTGCGGTACCATTATGAACGTTAAAGAGGGA

P.maritimus_dsAQP1         ------------------------------------------------------------
P.citri_AQP1               ATTTTAACCGTTCAAGTAGCCTTAACTTTCGGCCTATGTATCGCAGTTATGGCACAAAGT

P.maritimus_dsAQP1         ---------------------------------GCGGTTACGCTGAGTTTTCTGGTTGTT
P.citri_AQP1               ATCGGCCATGTGAGTGGATGTCACATAAATCCTGCGGTTACGCTCAGTTTTCTGGTTGTT
                                                            *********** ***************

P.maritimus_dsAQP1         GGTAAATGCTCGATTCTGAAATCGTTGTGTTACATTGTTTTACAATGCGCTGGCGCTACA
P.citri_AQP1               GGTAAATGCTCGATTCTGAAATCGTTATGTTACATTGTTTTACAATGCGCTGGAGCTGCA
                           ************************** ************************** *** **

P.maritimus_dsAQP1         GCTGGGTTTTATGTTTTAACTCTTCTGACCCCACAAGGCATTCTAACAGCTAATAAAAAC
P.citri_AQP1               GCTGGGTTTTATGTTTTACTTCTCGTGACTCCTCATGCGACCGAAGCTTCGTCCAATAAC
                           ******************  ***  **** ** ** *  *    * *  *    ** ***

P.maritimus_dsAQP1         CTCGGAAATACATTCTTAGGACCGAAAGTTACACCTGTACAAGGGCTCGGGATCGAGATT
P.citri_AQP1               CTCGGAAATACATCTTTAGGAAAAAATGTCACACCTTCACAAGGATTGGTAGTCGAGATC
                           *************  ******   ** ** ******  ******  * *   ******* 

P.maritimus_dsAQP1         ATTGCCACGTTTTTATTATGCTTCGTCATTCATTCAGTCTGCG-----------------
P.citri_AQP1               ATCGCAACATTTTTATTATGCTTCGTAATTCATTCGGTTTGCGATGAAAGGAGATCAGAT
                           ** ** ** ***************** ******** ** ****

P.maritimus_dsAQP1         ------------------------------------------------------------
P.citri_AQP1               GTGAAAATGATTGCTCCTTTATTGATTGGAATTTCTGCCGTCGTTTGCCATTTATTCGCA

P.maritimus_dsAQP1         ------------------------------------------------------------
P.citri_AQP1               ATTGATTACACTGGTTCGAGTTTGAATCCTGCTCGTAGTTTTGGCCCTACTGTTGTTTAT

P.maritimus_dsAQP1         ------------------------------------------------------------
P.citri_AQP1               GGCAAGTGGACCAATCATTGGGTTTACTGGGCTGGGCCTATAATCGGAGGTTGCGTCGCG

P.maritimus_dsAQP1         ------------------------------------------------------------
P.citri_AQP1               TCGATTGTGTACAAGCTGTTATTTCAGGTACGTAAAGGAGAAGAAGAAACGAGTTCATAC

P.maritimus_dsAQP1         ----------
P.citri_AQP1               GATTTTTCAT
```

Fig. S5(B)

```
P.maritimus_dsAQP1        ------------------------------------------------------------
P.ficus_AQP1              TGCTTTTCTTCGGCTGCGGTACCATTATGAACGTTAACAAAGACATTTTAACCGTTCAAG


P.maritimus_dsAQP1        ------------------------------------------------------------
P.ficus_AQP1              TAGCCTTAACTTTTGGCCTATGTATCGCAGTTATGGCACAGAGTATCGGCCATGTGAGTG


P.maritimus_dsAQP1        -----------------GCGGTTACGCTGAGTTTTCTGGTTGTTGGTAAATGCTCGATTC
P.ficus_AQP1              GATGTCACATAAATCCTGCGGTTACGCTCAGTTTTCTGATTGTTGGTAAATGCTCGATTC
                                           *********** ********* *********************

P.maritimus_dsAQP1        TGAAATCGTTGTGTTACATTGTTTTACAATGCGCTGGCGCTACAGCTGGGTTTTATGTTT
P.ficus_AQP1              TGAAATCGTTATGTTACATTGTTTTACAATGCGCTGGAGCTGCAGCTGGGTTTTATGTTT
                          ********** ************************** *** ******************

P.maritimus_dsAQP1        TAACTCTTCTGACCCCACAAGGCATTCTAACAGCTAATAAAAACCTCGGAAATACATTCT
P.ficus_AQP1              TACTTCTCGTGACCCCTCATGCTACCGAAGCTTCGTCCAAAAACCTCGGAAATACATCTT
                          **  ***  ******* ** *  *    * *  *    *******************  *

P.maritimus_dsAQP1        TAGGACCGAAAGTTACACCTGTACAAGGGCTCGGGATCGAGATTATTGCCACGTTTTTAT
P.ficus_AQP1              TAGGAATTAATGTCACACCTTCACAAGGATTGGTAGTCGAGATCATCGCAACATTTTTAT
                          *****   ** ** ******  ******  * *   ******* ** ** ** *******

P.maritimus_dsAQP1        TATGCTTCGTCATTCATTCAGTCTGCG---------------------------------
P.ficus_AQP1              TATGCTTCGTAATTCATTCGGTTTGCGATGAAAGGAGATCAGATGTGAAAATGATTGCTC
                          ********** ******** ** ****

P.maritimus_dsAQP1        ------------------------------------------------------------
P.ficus_AQP1              CTTTATTGATTGGAATTTCTGCCGTCGTTTGCCATTTATTCGCAATTGATTACACTGGTT


P.maritimus_dsAQP1        ------------------------------
P.ficus_AQP1              CGAGTTTGAATCCTGCTCGTAGTTTTGGCC
```

Fig. S5(C)

```
P.maritimus_dsNUC1      -----------------------------------------------CTTGAT-------
P.citri_NUC1            ATGAGTCACAATATTTTCACTATGATCAACGTGCTCGCTTCATTGTTCTTGGTATTTTTC
                                                                       **** *

P.maritimus_dsNUC1      ---------------------GAGTGCA--------------------------------
P.citri_NUC1            ACCATAACAGAATTACACGCCGAATGTAGCTTCTCATTAAACGAACACGTACCAAAAACC
                                             ** ** *

P.maritimus_dsNUC1      ------------------ACCCAAAATAACCAAATGCTCTACCCGAGCGAATCCAATACA
P.citri_NUC1            GCCATCATCACCAGCTTCACTCAACAAAACCAAATACTCTATCCGAACCATACCAATACA
                                          ** *** * ******** ***** **** * *  ********

P.maritimus_dsNUC1      ATCAGAATGCGTACCGGGACCCGGTTCAAGGTAAGCTGCGGCGATAAAGACTTCAAGAAA
P.citri_NUC1            CTCAAATTAGCCACTGGGGGCAGAATAAGGCTAACCTGCGGTGACCAATACTTCAAGAAA
                         *** * *    ** ***  * *  * * * *** ****** **  ** ***********

P.maritimus_dsNUC1      AAATTCAAAAAATCACCAAGAACCAAAGAAGTCCAAGCACGTTGCAATTCCAAAGACATA
P.citri_NUC1            AAATTCAAAAAATCGTCCAAAACCAAGGAGGTTGTAGCACGTTGCAATTCCAAAGACATC
                        **************  * * ****** ** **   ************************

P.maritimus_dsNUC1      ATCAATGTCGAAGGCGAACGAATACGTTTTCGAGAACTAGAATGTCAAAGTTTTCCAACA
P.citri_NUC1            GTCAACGTTGAAAATGAACGAATACGACTTCGAGAACTAGAATGTGAAGATTTCCCATCA
                         **** ** ***   *********** ***************** **  *** *** **

P.maritimus_dsNUC1      TCCAAACC-----------CCAAAAA----------------------------------
P.citri_NUC1            TCAAAGCCGCACAAGAAGACCGAAAAGAAATGCCATAAAAACAATACCTTGTTTGATATC
                        ** ** **           ** ****

P.maritimus_dsNUC1      ------------------------------------------------------------
P.citri_NUC1            GCTTTTGAAGTTCGCGGTGGTTCTTTGGACTCGATACGAGCATGTTTCGACGAACCGAAT

P.maritimus_dsNUC1      ------------------------------------------------------------
P.citri_NUC1            CAAGATTCCATTTACACGTGGTACGATACTTCGATGTTACCCACAGGACATCAGAGTAAC

P.maritimus_dsNUC1      ------------------------------------------------------------
P.citri_NUC1            GTCAAAAGACCCCAATTCGTGCACGATGATTTGTATAGATTTCCCGTCAGCGAAGTATAT

P.maritimus_dsNUC1      ------------------------------------------------------------
P.citri_NUC1            ACCGTTCATTATCAACACGATTGGTTTGCTAAATTGTTAAAATCGCAAAAAAAGGCTGAC

P.maritimus_dsNUC1      ------------------------------------------------------------
P.citri_NUC1            GAATATATTAAAAACGATGGTCAGCATTTTTTGTCCAGAGGTCATCTCACAGCAAAGGCT

P.maritimus_dsNUC1      ------------------------------------------------------------
P.citri_NUC1            GACATGGTGTATGGATCAGAGCAGTCGGCTACCTTCCATTATATCAATGTTGCACCTCAA

P.maritimus_dsNUC1      ------------------------------------------------------------
P.citri_NUC1            TGGCAATCTTTTAATGGTGGTAATTGGAATAGAGTAGAAGAAAGTGTCAGGAAAGAGATC

P.maritimus_dsNUC1      ------------------------------------------------------------
P.citri_NUC1            CAGAAGAAGGATAAAAGATACCGAGTTGTAACTGGAACCCACGGAATAGCCACACTACCC

P.maritimus_dsNUC1      ------------------------------------------------------------
P.citri_NUC1            GACGTAAACAACAACGAGCAAGAACTCTACCTTTACGCGGACGAGAACAAAAACCCCTTA
```

*P.maritimus*_ds*NUC1* --
P.citri_NUC1 CTCAAAGTGCCTAAGCTTTTCTGGAAACTAGTCTACGACATGAATCGAGAAGAAGGAGTC

*P.maritimus*_ds*NUC1* --
P.citri_NUC1 GTTATCATCGGTGTAAATAACCCATACCTGAAGAAAATCCCCTCGGATTACATAATTTGC

*P.maritimus*_ds*NUC1* --
P.citri_NUC1 AAAGATATCTGCAACAAAATCCGATGGTTGCCCGAGTTCAATAAAGATAACAGCAAAGGA

*P.maritimus*_ds*NUC1* --
P.citri_NUC1 TACATATACTGCTGCGAAATGGACGAATTTCTCAAAGTGACCGGTTTCGATAAATCATTC

*P.maritimus*_ds*NUC1* ---------
P.citri_NUC1 ATGAATTAA

Fig. S5(D)

```
P.maritimus_dsNUC1          ---------------------------------------------CTTGATGAGTGCAAC
P.ficus_NUC1                AATGCGAGTGCTTCTCATTAACGAACACGTTCCAAAAACCGCCATCATAACCAGCATCAC
                                                                         * * *  **    **

P.maritimus_dsNUC1          CCAAAATAACCAAATGCTCTACCCGAGCGAATCCAATACAATCAGAATGCGTACCGGGAC
P.ficus_NUC1                TCAACAAAACCAAATACTTTATCCGAACACAACCAATACGCTCAAATTAGCCACTGGAGG
                             *** * ******** ** ** **** *  * *******  *** * *    ** **   

P.maritimus_dsNUC1          CCGGTTCAAGGTAAGCTGCGGCGATAAAGACTTCAAGAAAAAATTCAAAAAATCACCAAG
P.ficus_NUC1                CAGGATAAGGCTAACCTGCGGTGACAAATACTTCAAGAAAAAATTTAAAAAATCGTCAAA
                            * ** * * * *** ****** ** *** **************** ********  *** 

P.maritimus_dsNUC1          AACCAAAGAAGTCCAAGCACGTTGCAATTCCAAAGACATAATCAATGTCGAAGGCGAACG
P.ficus_NUC1                AACCAAGGAGGTTGTGGCACGTTGCAATTCCAAAGACGTCGTCAACGTTGAAAATGAACG
                            ****** ** **    ********************* *  **** ** ***   *****

P.maritimus_dsNUC1          AATACGTTTTCGAGAACTAGAATGTCAAAGTTTTCCAACATCCAAACC-----------C
P.ficus_NUC1                AATACGACTTCGAGAACTAGAATGTGAAGATTTCCCATCATCAAAGCCGCACAAGAAGAC
                            ******  ***************** **  *** *** **** ** **           *

P.maritimus_dsNUC1          CAAAAA------------------------------------------------------
P.ficus_NUC1                CGAAAAGAAATGCCATCAAAACAATACCTTGTTTGATATCGCTTTTGAAGTTCGTGGTGG
                            * ****

P.maritimus_dsNUC1          ------------------------------------------------------------
P.ficus_NUC1                TTCTTTGGACTCGATACGAGCATGTTTTGACGAACCGAATCAAGATTCGATTTACACGTG

P.maritimus_dsNUC1          ------------------------------------------------------------
P.ficus_NUC1                GTACGATACGTCGATGTTACCCACAGGACATCAGAGTAACGTCAAAAGACCCCAATTCGC

P.maritimus_dsNUC1          ------------------------------------------------------------
P.ficus_NUC1                GCACGATGATTTGTATAGATTTCTTGTCTACGAAGCATATACCATTAATCATCAACGTGA

P.maritimus_dsNUC1          ------------------------------------------------------------
P.ficus_NUC1                TCAGTTTGCTAATTTGTTACAATCGAAAGAAAAGGCTGATGAATATATTAAAAACGATGG

P.maritimus_dsNUC1          ------------------------------------------------------------
P.ficus_NUC1                CAAGCATTTTTTGTCCAGAGGTCATCTCACAGCAAAAGCTGACATGGTGTATGGATCGGA

P.maritimus_dsNUC1          ------------------------------------------------------------
P.ficus_NUC1                GCAGTCGGCTACTTTTCATTATATCAATGTCGCACCTCAATGGCAATGTTTTAATGATGG

P.maritimus_dsNUC1          ------------------------------------------------------------
P.ficus_NUC1                TAATTGGAATAAAGTAGAAGATAGCGTCAGGAAAGAGATCCAGAAGAAGGATAAAAGATA

P.maritimus_dsNUC1          ------------------------------------------------------------
P.ficus_NUC1                CCGAGTCGTAACTGGAACCCACGGAATAGCCACACTACCCGACGTAAACAACAACGAGCA

P.maritimus_dsNUC1          ------------------------------------------------------------
P.ficus_NUC1                AGAACTCTACCTTTACGAGGACGAGAACAAGAAACCCTTACTCAAAGTGCCTAAGCTTTT

P.maritimus_dsNUC1          ---------------------------
P.ficus_NUC1                TTGGAAACTAGTCTACGACCTGATCGG
```
