# Supplementary material for: Evaluation of RNA Interference for Control of the Grape Mealybug Pseudococcus maritimus (Hemiptera: Pseudococcidae)
Source: Insects. 2020 Oct 28;11(11):739. doi: 10.3390/insects11110739 (PMC7692628; doi:10.3390/insects11110739)
Supplement: Supplementary file 1 [file insects-11-00739-s001.zip › supplementary/Supp_methods_tables.docx]

**Phylogenetic analysis of candidate genes**

The protein sequences of aquaporins, glucosyl hydrolases of family 13 (GH-13) and nucleases were obtained from publicly available insect genome data in Aphidbase (<https://bipaa.genouest.org/is/aphidbase/>), Vectorbase (<https://www.vectorbase.org/>) and NCBI (<https://blast.ncbi.nlm.nih.gov/Blast.cgi>). For the mealybug *Planococcus citri*, we used transcriptome data (SRP021919] [1], specifically previously collated sequences for aquaporins and GH-13 genes [2] and to identify nuclease sequence the transciptome data was first assembled using TRINITY package with default settings [3]. The assembled sequences were used to make a local database using NCBI command line and the nuclease sequence was searched using *A. pisum* nuclease sequence (Accession# ACYPI008471) as the query. The secretion of the identified nuclease was confirmed using signal-4.0 [4]. Retrieved protein sequences were identified by the presence of MIP superfamily domain (cl00200) for aquaporins, α-amylase domain (cd11328) for glucosyl hydrolases and of DNA/RNA non-specific endonuclease domain (cl00089) for nucleases, using the CDD platform in NCBI.

The selected protein sequences were aligned in ClustalW [5] and trimmed using Trimal (v. 1.3) [5] with gap threshold of 0.25 on Phylemon2 platform (<http://phylemon.bioinfo.cipf.es/utilities.html> ) [6]. The best models of protein evolutionwere selected using Prottest2.4, yielding LG+I+G+F, LG+I+G, and WAG+G+F for aquaporins, sucrases and nucleases, respectively. MrBayes 3.2.6 [7] was used to construct Bayesian phylogeny, and iqtree (v1.6.12 ) [8] was used to determine Maximum Likelihood phylogeny. Bayesian inference used two runs with four chains per run and analysis was conducted for 1 million generations with the standard deviation of split frequencies less than 0.05. Convergence was confirmed with TRACER v1.6 (beast.bio.ed.ac.uk/Tracer) assuming a burn-in of 10% of generations. The initial 10% of generations were removed and phylogenies sampled in the remaining generations were used to estimate a 50% majority-rule consensus tree. The maximum likelihood trees were constructed with 1000 replicates of ultrabootstrap [9] and the Shimodaira-Hasegawa-like approximate likelihood ratio test [10] with 1000 replicates.

**Supplementary Table S1. Primer sequences**

| Gene | Primer Direction | Primer binding sites (nt number of pea aphid transcript) | Primer sequence (5`-3`) |
| --- | --- | --- | --- |
| 1. Sanger sequencing | | | |
| *Aquaporin-1* | Forward-1 | 45-63 | CATGCTATGCGCCGAATTT |
|  | Reverse-1 | 619-638 | CCAGCCCAGTAAACCCAATG |
| *Sucrase-1* | Forward-1 | 553-572 | GCTTGGGAATGGAATGAAGA |
|  | Reverse-1 | 1084-1100 | CCWATCACCCAGTTDGCC |
| *Sucrase-4* | Forward-1 | 85-110 | GATACTACATTAGATTGGTGGCAGAC |
|  | Reverse-1 | 580-604 | CTGGTTGTTTTCTGGCGAACTGATG |
| *Nuclease-1* | Forward-1 | 67-90 | GTTCTTGGTATTTTTCACCATAAC |
|  | Reverse-1 | 490-509 | GGTTCGTCGAAACATGCTCG |
|  | Forward-1 | 67-90 | GTTCTTGGTATTTTTCACCATAAC |
|  | Reverse-2 | 1065-1086 | CAGGTATGGGTTATTTACACCG |
|  | Forward-3 | 340-359 | GTCGAAGGCGAACGAATACG |
|  | Reverse-3 | 548-568 | GATGTCCTGTTGGTAGCATCG |
|  | Forward-4 | 383-403 | GTTTTCCAACATCGAAACCCC |
|  | Reverse-4 | 1018-1042 | GAACCATGTCGTAGACTAGTTTTCC |
| *β-tubulin* | Forward | 34-50 | TGCGGAAAYCARATYGG |
|  | Reverse | 676-695 | TTKARGTCVCCGTABKTBGG |

| 1. dsRNA primers | | | |
| --- | --- | --- | --- |
| Gene name | Primer | Amplified basepair number | Primer sequence |
| *Aquaporin-1* | Forward | 250 | **TAATACGACTCACTATAGG**GCGGTTACGCTGAGTTTTCT |
|  | Reverse |  | **TAATACGACTCACTATAGG**CGCAGACTGAATGAATGACG |
| *Sucrase-1* | Forward | 240 | **TAATACGACTCACTATAGG**TTTGGATGAACCACTTTCAGG |
|  | Reverse |  | **TAATACGACTCACTATAGG**AAAGGTAAATGAGCTCCGGG |
| *Nuclease-1* | Forward | 250 | **TAATACGACTCACTATAGG**CTTGATGAGTGCAACCCAAA |
|  | Reverse |  | **TAATACGACTCACTATAGG**TTTTTGGGGTTTGGATGTTG |
| c) qRT-PCR | | | |
| *Aquaporin-1* | Forward | 106 | GGCTGCGGTACCATTATGAA |
|  | Reverse |  | TCACATGGCCGATACTTTGG |
| *Sucrase-1* | Forward | 70 | TGGTTGTGCTACGAAGAAATG |
|  | Reverse |  | CAATCCATCGATACCCTTGG |
| *Sucrase-4* | Forward | 99 | GGAATCGAAGAGCAAGCTAA |
|  | Reverse |  | ATCGTAGCCCATATCAACCA |
| *Nuclease-1* | Forward | 100 | TTCGTCGATGCTACCAACAG |
|  | Reverse |  | ACTCTTGTACACCTCGTCGA |
| *β-tubulin* | Forward | 64 | GCCGGACCTTTCGGTCAAAT |
|  | Reverse |  | AATTCGGCACCTTCGGTGTA |

**Supplementary Table S2. PCR amplification conditions to amplify different segments of the genes**

| Gene | Primer combination | Initial denaturation | Denaturation | Annealing | Elongation | Final elongation |
| --- | --- | --- | --- | --- | --- | --- |
| *Aquaporin-1* | Forward-1 + Reverse-1 | 95^˚^C for 3 minutes | 95^˚^C for 45 seconds | 53.1^˚^C for 45 seconds | 72^˚^C for 2 minutes | 72^˚^C for 5 minutes |
| *Sucrase-1* | Forward-1 + Reverse-1 | 95^˚^C for 3 minutes | 95^˚^C for 45 seconds | 53.1^˚^C for 45 seconds | 72^˚^C for 2 minutes | 72^˚^C for 5 minutes |
| *Sucrase-4* | Forward-1 + Reverse-1 | 95^˚^C for 3 minutes | 95^˚^C for 30 seconds | 55^˚^C for 45 seconds | 72^˚^C for 2 minutes | 72^˚^C for 5 minutes |
| *Nuclease-1* | Forward-1 + Reverse-1 | 95^˚^C for 3 minutes | 95^˚^C for 30 seconds | 45^˚^C for 45 seconds | 72^˚^C for 2 minutes | 72^˚^C for 5 minutes |
|  | Forward-1 + Reverse-2 | 95^˚^C for 3 minutes | 95^˚^C for 30 seconds | 50^˚^C for 45 seconds | 72^˚^C for 2 minutes | 72^˚^C for 5 minutes |
|  | Forward-3 + Reverse-3 | 95^˚^C for 3 minutes | 95^˚^C for 30 seconds | 50^˚^C for 45 seconds | 72^˚^C for 2 minutes | 72^˚^C for 5 minutes |
|  | Forward-4+ Reverse-4 | 95^˚^C for 3 minutes | 95^˚^C for 30 seconds | 50^˚^C for 45 seconds | 72^˚^C for 2 minutes | 72^˚^C for 5 minutes |
| *Β-tubulin* | Forward  Reverse | 95^˚^C for 3 minutes | 95^˚^C for 30 seconds | 50^˚^C for 45 seconds | 72^˚^C for 2 minutes | 72^˚^C for 5 minutes |

**Supplementary Table S3** : Effect of RNAi treatments on *P. maritimus* survival

| *AQP-1* | | |
| --- | --- | --- |
| Treatment | Regression coefficient | Pr(>\|t\|) |
| Diet | 0.944±0.3894 | 0.3453 |
| ds*NUC*+ds*GFP* | 0.895±0.3897 | 0.3705 |
| ds*AQP+*ds*SUC* | -0.444±0.3645 | 0.6567 |
| ds*NUC*+ds*AQP*+ds*SUC* | **-1.650±0.3532*** | **0.0990** |

ds*GFP* was used as the intercept and other treatments were compared to the intercept.

P values below 0.1 are bolded and marked with single asterisk.

**Supplementary Table S4** : Effect of RNAi treatments on expression of various *P. maritimus* genes

| *AQP-1* | | |
| --- | --- | --- |
| Treatment | Regression coefficient | Pr(>\|t\|) |
| Diet | 0.638±0.5955 | 0.5330 |
| ds*NUC*+ds*GFP* | -0.225±0.5955 | 0.8253 |
| ds*AQP+*ds*SUC* | -0.978±0.5955 | 0.3435 |
| ds*NUC*+ds*AQP*+ds*SUC* | **-2.462±0.5955**** | **0.0264** |
| *SUC-1* | | |
| Diet | 0.444±1.27713 | 0.6631 |
| ds*NUC*+ds*GFP* | -0.0.76±1.27713 | 0.9402 |
| ds*AQP+*ds*SUC* | -1.375±1.27713. | 0.1893 |
| ds*NUC*+ds*AQP*+ds*SUC* | **-2.054±1.27713*** | **0.0578** |
| *NUC1* | | |
| Diet | 1.245±1.034 | 0.2321 |
| ds*NUC*+ds*GFP* | -0.023±1.034 | 0.9820 |
| ds*AQP+*ds*SUC* | -0.822±1.034. | 0.4239 |
| ds*NUC*+ds*AQP*+ds*SUC* | **-1.914±1.034*** | **0.0749** |

ds*GFP* was used as the intercept and other treatments were compared to the intercept.

P values below 0.05 are bolded and marked with double asterisk, and P values below 0.1 are bolded and marked with single asterisk.

**References**

1. Husnik, F.; Nikoh, N.; Koga, R.; Ross, L.; Duncan, R.P.; Fujie, M.; Tanaka, M.; Satoh, N.; Bachtrog, D.; Wilson, A.C.C.; et al. XHorizontal gene transfer from diverse bacteria to an insect genome enables a tripartite nested mealybug symbiosis. *Cell* **2013**, *153*, 1567, doi:10.1016/j.cell.2013.05.040.

2. Jing, X.; White, T.A.; Luan, J.; Jiao, C.; Fei, Z.; Douglas, A.E. Evolutionary conservation of candidate osmoregulation genes in plant phloem sap-feeding insects. *Insect Mol. Biol.* **2016**, *25*, 251–258, doi:10.1111/imb.12215.

3. Haas, B.J.; Papanicolaou, A.; Yassour, M.; Grabherr, M.; Philip, D.; Bowden, J.; Couger, M.B.; Eccles, D.; Li, B.; Macmanes, M.D.; et al. *reference generation and analysis with Trinity*; 2014; Vol. 8; ISBN 3942667509.

4. Petersen, T.N.; Brunak, S.; Von Heijne, G.; Nielsen, H. SignalP 4.0: Discriminating signal peptides from transmembrane regions. *Nat. Methods* **2011**, *8*, 785–786, doi:10.1038/nmeth.1701.

5. Larkin, M.A.; Blackshields, G.; Brown, N.P.; Chenna, R.; Mcgettigan, P.A.; McWilliam, H.; Valentin, F.; Wallace, I.M.; Wilm, A.; Lopez, R.; et al. Clustal W and Clustal X version 2.0. *Bioinformatics* **2007**, *23*, 2947–2948, doi:10.1093/bioinformatics/btm404.

6. Sánchez, R.; Serra, F.; Tárraga, J.; Medina, I.; Carbonell, J.; Pulido, L.; De María, A.; Capella-Gutíerrez, S.; Huerta-Cepas, J.; Gabaldón, T.; et al. Phylemon 2.0: A suite of web-tools for molecular evolution, phylogenetics, phylogenomics and hypotheses testing. *Nucleic Acids Res.* **2011**, *39*, 470–474, doi:10.1093/nar/gkr408.

7. Ronquist, F.; Teslenko, M.; Van Der Mark, P.; Ayres, D.L.; Darling, A.; Höhna, S.; Larget, B.; Liu, L.; Suchard, M.A.; Huelsenbeck, J.P. Mrbayes 3.2: Efficient bayesian phylogenetic inference and model choice across a large model space. *Syst. Biol.* **2012**, *61*, 539–542, doi:10.1093/sysbio/sys029.

8. Minh, B.Q.; Schmidt, H.A.; Chernomor, O.; Schrempf, D.; Woodhams, M.D.; von Haeseler, A.; Lanfear, R. IQ-TREE 2: New models and efficient methods for phylogenetic inference in the genomic era. *Mol. Biol. Evol.* **2020**, 6–10, doi:10.1093/molbev/msaa015.

9. Hoang, D.T.; Chernomor, O.; von Haeseler, A.; Minh, B.Q.; Vinh, L.S. UFBoot2: Improving the Ultrafast Bootstrap Approximation. Molecular biology and evolution. *Mol. Biol. Evol.* **2018**, *35*, 518–522, doi:10.5281/zenodo.854445.

10. Guindon, S.; Dufayard, J.F.; Lefort, V.; Anisimova, M.; Hordijk, W.; Gascuel, O. New algorithms and methods to estimate maximum-likelihood phylogenies: Assessing the performance of PhyML 3.0. *Syst. Biol.* **2010**, *59*, 307–321, doi:10.1093/sysbio/syq010.
